# Mendelian randomization study of inflammatory bowel disease and bone mineral density

**DOI:** 10.1186/s12916-020-01778-5

**Published:** 2020-11-10

**Authors:** Fashuai Wu, Yu Huang, Jialu Hu, Zengwu Shao

**Affiliations:** 1 Department of Orthopaedics, Union Hospital, Tongji Medical College, Huazhong University of Science and Technology, Wuhan, 430022 China; 2 Department of Otorhinolaryngology, The Third Hospital of Wuhan City, Wuhan, 430070 China; 3 School of Computer Science, Northwestern Polytechnical University, West Youyi Road 127, Xi’an, 710072 China

**Keywords:** Two-sample Mendelian randomization, Inflammatory bowel disease, Ulcerative colitis, Crohn’s disease, Bone mineral density, Osteoporosis

## Abstract

**Background:**

Recently, the association between inflammatory bowel disease (including ulcerative colitis and Crohn’s disease) and BMD has attracted great interest in the research community. However, the results of the published epidemiological observational studies on the relationship between inflammatory bowel disease and BMD are still inconclusive. Here, we performed a two-sample Mendelian randomization analysis to investigate the causal link between inflammatory bowel disease and level of BMD using publically available GWAS summary statistics.

**Methods:**

A series of quality control steps were taken in our analysis to select eligible instrumental SNPs which were strongly associated with exposure. To make the conclusions more robust and reliable, we utilized several robust analytical methods (inverse-variance weighting, MR-PRESSO method, mode-based estimate method, weighted median, MR-Egger regression, and MR.RAPS method) that are based on different assumptions of two-sample MR analysis. The MR-Egger intercept test, Cochran’s *Q* test, and “leave-one-out” sensitivity analysis were performed to evaluate the horizontal pleiotropy, heterogeneities, and stability of these genetic variants on BMD. Outlier variants identified by the MR-PRESSO outlier test were removed step-by-step to reduce heterogeneity and the effect of horizontal pleiotropy.

**Results:**

Our two-sample Mendelian randomization analysis with two groups of exposure GWAS summary statistics and four groups of outcome GWAS summary statistics suggested a definitively causal effect of genetically predicted ulcerative colitis on TB-BMD and FA-BMD but not on FN-BMD or LS-BMD (after Bonferroni correction), and we merely determined a causal effect of Crohn’s disease on FN-BMD but not on the others, which was somewhat inconsistent with many published observational researches. The causal effect of inflammatory bowel disease on TB-BMD was significant and robust but not on FA-BMD, FN-BMD, and LS-BMD, which might result from the cumulative effect of ulcerative colitis and Crohn’s disease on BMDs.

**Conclusions:**

Our Mendelian randomization analysis supported the causal effect of ulcerative colitis on TB-BMD and FA-BMD. As to Crohn’s disease, only the definitively causal effect of it on decreased FN-BMD was observed. Updated MR analysis is warranted to confirm our findings when a more advanced method to get less biased estimates and better precision or GWAS summary data with more ulcerative colitis and Crohn’s disease patients was available.

## Background

The incidence of aging-related disorders has dramatically increased in modern society for improved healthcare, socio-economic, and lifestyle changes which greatly increased life expectancy [1]. Osteoporosis is a common, aging-related systemic skeletal disease characterized by decreased bone strength, micro-architectural deterioration of bone tissue, and consequent increased fracture risk [2, 3]. It is clinically diagnosed largely through measurement of bone mineral density (BMD) at central sites (the lumbar spine and the proximal femur) and peripheral sites (including the distal forearm) as examined by dual-energy X-ray absorptiometry (DXA) [2, 4]. In the USA, the prevalence of osteoporosis is estimated to increase to more than14 million cases in 2020, and the burden is projected to increase to exceed 3 million fractures and $25.3 billion each year by 2025 [5]. Clearly, the severe clinical and economic consequences of osteoporosis urgently call for a concerted effort to identify the risk factors causing osteoporosis and assess patients at risk to allow for prevention and early intervention when appropriate. The etiology of osteoporosis is not well understood. It is well recognized that increasing age, female gender, and a wide range of clinical factors, medical factors, behavior factors, nutritional factors, and genetic factors are associated with the disease [2, 6–9]. Many studies demonstrated that the potential risk factors including cigarette smoking, heavy alcohol intake, caffeine intake, glucocorticoid therapy, low body mass index (BMI), physical inactivity, gastrointestinal diseases, hematologic disorders, and calcium and vitamin D deficiency may contribute to low BMD and fractures [2, 10, 11].

Inflammatory bowel disease (IBD), which includes ulcerative colitis (UC) and Crohn’s disease (CD), is a chronic, relapsing inflammatory condition of the gastrointestinal tract [12]. It affects more than 2.5 million people in Europe, with increasing prevalence in Asia and developing countries [13]. Recently, the association between IBD and BMD has gained great interest. However, available epidemiological evidences on the effects of IBD on the level of BMD are inconclusive. A population-based prospective study containing 60 UC patients and 60 CD patients demonstrated that only minor changes in BMD were observed in both CD and UC patients during a 2-year period [14]. Another study found that steroid-naive young male patients with IBD had lower bone density values than healthy controls [15]. Some studies have revealed that decreased BMD in individuals with IBD was related to corticosteroid use but not the disease itself [16]. And some studies concluded that BMD is reduced in patients with CD but not in patients with UC [17–19]. Given that the studies, which have drawn inconsistent conclusions, were either based on limited samples or only explored the correlations between IBD (including UC and CD) and BMD and osteoporosis, and the epidemiological observational studies may be subjected to confounding factors and reverse causality [20]. A study, like randomized controlled trials (RCTs), directly inferring the causal relationship between IBD and BMD and osteoporosis is helpful for the prevention and early intervention of osteoporosis and consequent fractures in high-risk populations. However, RCTs are difficult or impractical to perform for they are expensive, labor resource-intensive, time-consuming, and ethical limitations. As an alternative, Mendelian randomization (MR), mimic the design of RCT, is a popular yet more convenient technique to test the causality between an exposure (IBD) and an outcome (BMD or osteoporosis) [20].

Two-sample MR is a technique, using germline genetic variants as instrument variables (IV) for exposure to study the causal relations between the exposure phenotype and the outcome phenotype. It enables the use of publically available results from very large genome-wide association studies (GWAS) for both risk factor “exposures” and disease “outcomes” and overcomes the typical pitfalls present in observational studies. In order to obtain unbiased estimates, MR need to fulfill three key assumptions: IV1—genetic variants used in the analysis should be significantly associated with the exposure; IV2—genetic variants extracted as instrument variables for exposure are independent of confounding factors that are associated with the selected exposure and outcome; and IV3—the genetic variants affects the outcome only through the exposure and not via other biological pathways (i.e., no horizontal pleiotropic effect) [21]. Here, a two-sample Mendelian randomization analysis was performed to investigate the causal link between IBD (including UC and CD) and decreased BMD, in which we used the summary statistics from GWAS data of IBD (including UC and CD) and BMDs (including total body BMD (TB-BMD), femoral neck BMD (FN-BMD), lumbar spine BMD (LS-BMD), and forearm BMD (FA-BMD)).

## Methods

### IBD and BMD GWAS summary statistics

To obtain a more comprehensive and reliable conclusion of the causal link between IBD and BMDs, we selected the largest GWAS published to date for IBD including UC and CD [22]. Another study with a larger GWAS of IBD was also included for replication purposes [23]. Full summary statistics for the IBD (unit, logOR) GWAS are available for download from the International IBD Genetics Consortium’s website at https://www.ibdgenetics.org/downloads.html. The datasets used for replication are available at https://gwas.mrcieu.ac.uk/datasets/. The femoral neck, lumbar spine, and forearm are the three common skeletal sites of postmenopausal women and men who are 50 years or older for measurement of BMD based on DXA. Total body BMD (TB-BMD) GWAS summary data is used to estimate the general effect of IBD on whole-body BMD. TB-BMD measurement is the most appropriate method for an unbiased assessment of BMD variation in the same skeletal site from childhood to old age [24]. GWAS summary statistics for BMDs (unit, g/cm^2^) was downloaded from the GEnetic Factors for OSteoporosis Consortium website (GEFOS, http://www.gefos.org/). We also could download GWAS summary statistics of IBD and BMD from the publicly available GWAS catalog website (https://www.ebi.ac.uk/gwas/downloads/summary-statistics) or IEU GWAS database (https://gwas.mrcieu.ac.uk/datasets/). The corresponding effect estimates of SNP on IBD (including UC and CD) and BMD had been adjusted for many principal components. The diagnosis of IBD was based on accepted radiologic, endoscopic, and histopathologic criteria. Measurement of BMD was recommended utilizing dual-energy X-ray absorptiometry.

The summary statistics of the largest GWAS published to date for IBD (*N* = 12,882 cases, 21,770 controls), UC (*N* = 6968 cases, 20,464 controls), and CD (*N* = 5956 cases, 14,927 controls) was obtained from the International IBD Genetics Consortium [22]. All participants were of European ancestry.

Summary statistics of a combined analysis including 38,565 IBD cases and 37,747controls and immunochip-wide association analyses with UC (*N* = 10,920 cases, 15,977 controls) and CD (*N* = 14,763 cases, 15,977 controls) were included for replication purposes [23]. To reduce the possibility of population stratification, all participants were of European ancestry. GWAS summary statistics were downloaded from https://gwas.mrcieu.ac.uk/datasets/.

Three separate GWAS summary statistics of European participants’ femoral neck bone mineral density (FN-BMD, *n* = 32,735), lumbar spine bone mineral density (LS-BMD, *n* = 28,498), and forearm bone mineral density (FA-BMD, *n* = 8143) were downloaded from GEFOS; it is the largest GWAS on DXA-measured BMD to date [8].

A meta-analysis comprising 56,284 individuals of European ancestry was performed to investigate the genetic determinants of total body bone mineral density (TB-BMD) [24]. The meta-analyzed effect size estimates were used in this study. The GWAS summary statistic of TB-BMD was downloaded from the GEFOS website.

### Genetic instrumental variables

From the GWAS summary data of IBD including UC and CD, we conducted a series of quality control steps to select eligible instrumental SNPs. Firstly, we extracted SNPs associated with IBD with genome-wide significance (*P* < 5 × 10^−8^). Secondly, it is important to ensure that all the instrumental SNPs for the exposure are not in linkage disequilibrium (LD), since instrumental SNPs in strong LD may cause biased results. In this study, we performed the clumping process (*R*^2^ < 0.001, window size = 10,000 kb) with the European samples from the 1000 genomes project which were used to estimate LD between SNPs. Among those pairs of SNPs that had LD *R*^2^ above the specified threshold (*R*^2^ = 0.001) only the SNP with the lower *P* value would be retained. SNPs absent from the LD reference panel were also removed. Thirdly, SNPs with minor allele frequency (MAF) < 0.01 were removed. Fourthly, extracting data for the above-selected SNPs from the outcome trait (BMDs) GWAS summary. By default, if a particular requested SNP was not present in the outcome GWAS, then a SNP (proxy) that was in LD with the requested SNP (target) would be searched for instead. LD proxies were defined using 1000 genomes of European sample data. The effect of the proxy SNP on the outcome was returned, along with the proxy SNP, the effect allele of the proxy SNP, and the corresponding allele (in phase) for the target SNP. Fifthly, the effect of ambiguous SNPs with non-concordant alleles (e.g., A/G vs. A/C) and palindromic SNPs with an ambiguous strand (i.e., A/T or G/C) was corrected or the ambiguous and palindromic SNPs were directly excluded from the above-selected instrument SNPs in harmonizing process to ensure that the effect of a SNP on the exposure, and the effect of that same SNP on the outcome, corresponds to the same allele. These stringently selected SNPs were used as the instrumental variables for subsequent two-sample MR analysis.

According to the assumptions of MR analysis, the selected instrumental SNPs should strongly associate with exposure. To test whether there was a weak instrumental variable bias, namely genetic variants selected as instrumental variables had a weak association with exposure, we calculated the *F* statistic (*F* = *R*^2^(*n* − *k* − 1)/*k*(1 − *R*^2^); *R*^2^, variance of exposure explained by selected instrumental variables, and we got the value of *R*^2^ in MR Steiger directionality test; *n*, sample size; and *k*, number of instrumental variables). If the *F* statistic is much greater than 10 for the instrument-exposure association, the possibility of weak instrumental variable bias is small [25].

### Mendelian randomization estimates

MR analysis uses genetic variants as instrumental variables to estimate the causative effect of exposure variables on an outcome. In the study, we combined the summary statistics (*β* coefficients and standard errors) to estimate the causal associations between IBD (including UC and CD) and BMDs (including TB-BMD, FN-BMD, LS-BMD, and FA-BMD) using different methods. Since it is unlikely that all genetic variants would be valid instrumental variables, several robust methods have been proposed. The methods which included inverse variance weighting (IVW), MR-Pleiotropy RESidual Sum and Outlier (MR-PRESSO) method, mode-based estimate (MBE) method, weighted median (WM), MR-Egger regression, and robust adjusted profile score (MR.RAPS) method were based on different assumptions.

The IVW method uses a meta-analysis approach to combine Wald estimates for each SNP (i.e., the *β* coefficient of the SNP for BMD divides by the *β* coefficient of the SNP for IBD) to get the overall estimates of the effect of IBD on BMD [26]. If there is no violation of the IV2 assumption (no horizontal pleiotropy), or the horizontal pleiotropy is balanced, an unbiased causal estimate can be obtained by IVW linear regression [27]. Fixed and random effects IVW approaches are available. If significant heterogeneity (*P* < 0.05) is observed, a random-effect IVW model is applied. MR-PRESSO is a method for the detection and correction of outliers in IVW linear regression. MR-PRESSO has three components, including (a) detection of horizontal pleiotropy (MR-PRESSO global test), (b) correction for horizontal pleiotropy via outlier removal (MR-PRESSO outlier test), and (c) testing of significant differences in the causal estimates before and after correction for outliers (MR-PRESSO distortion test). The MR-PRESSO outlier test requires that at least 50% of the variants are valid instruments, has balanced pleiotropy, and relies on the Instrument Strength Independent of Direct Effect (InSIDE) condition that instrument-exposure and pleiotropic effects are uncorrelated [28]. The mode-based method clusters the SNPs into groups basing on the similarity of causal effects and returns the causal effect estimate basing on the cluster that has the largest number of SNPs. The causal estimate from the mode-based estimator is unbiased if the SNPs contributing to the largest cluster are valid instruments even if the majority of instruments are invalid [29]. The median-based approach will provide an unbiased estimate of the causal effect in the presence of unbalanced horizontal pleiotropy even when up to 50% of SNPs are invalid IVs (e.g., due to pleiotropy) [30]. If there is a particular direction of the horizontal pleiotropic effect, then constraining the slope to go through zero will introduce bias. Egger regression which allows the intercept to pass through a value other than zero will relax the constraint. The MR-Egger regression, based on the assumption of InSIDE, performs a weighted linear regression of the outcome coefficients on the exposure coefficients [31]. Under the InSIDE assumption, it gives a valid test of the null causal hypothesis and a consistent causal effect estimate even when all the genetic variants are invalid IVs [31]. However, MR-Egger estimates may be inaccurate and can be strongly influenced by outlying genetic variants. The WM estimate which does not require the InSIDE assumption has been confirmed to have distinct superiorities over MR-Egger for its improved power of causal effect detection and lower type I error [30]. When the InSIDE assumption is valid and the percentage of horizontal pleiotropic variants is small (≤ 10%), the causal estimate of the MR-PRESSO outlier adjustment is less biased and has better precision (smaller standard deviation) than MR-Egger. However, when the percentage of horizontal pleiotropic variants is high (≥ 50%), the opposite is found [28]. The weighted median has less bias but also less precision in the causal estimate compared to the MR-PRESSO outlier test, particularly when the percentage of horizontal pleiotropic variants is < 50% [28]. Since we included many weak instrumental variables in the analyses, we carried out a recently proposed method called MR.RAPS to make our results more reliable [32]. This method is robust to both systematic and idiosyncratic pleiotropy and can give a robust inference for MR analysis with many weak instruments. It is able to correct for pleiotropy using robust adjusted profile scores and is recommended to routinely use the RAPS estimator in practice, especially if the exposure and the outcome are both complex traits.

If the estimates of different methods are inconclusive, the link between exposure and outcome phenotype with an adjusted *P* value < 0.05/5 = 0.01 (Bonferroni correction for multiple testing) is considered significant.

### Pleiotropy and sensitivity analysis

We conducted the MR-Egger regression to assess the potential pleiotropic effects of the SNPs used as IVs. The intercept term in MR Egger regression can be a useful indication of whether directional horizontal pleiotropy is driving the results of a MR analysis [33]. In MR-PRESSO analysis, it attempts to reduce heterogeneity in the estimate of the causal effect by removing SNPs that contribute to the heterogeneity disproportionately more than expected. The number of distributions in MR-PRESSO analysis was set to 1000. We used the IVW method and MR-Egger regression to detect heterogeneity. The heterogeneities were quantified by Cochran *Q* statistic; a *P* value of < 0.05 would be regarded as significant heterogeneity. Additionally, to identify potentially influential SNPs, we performed a “leave-one-out” sensitivity analysis to where the MR is performed again but leaving out each SNP in turn.

### Procedures of MR analysis

**Fig. 1:**
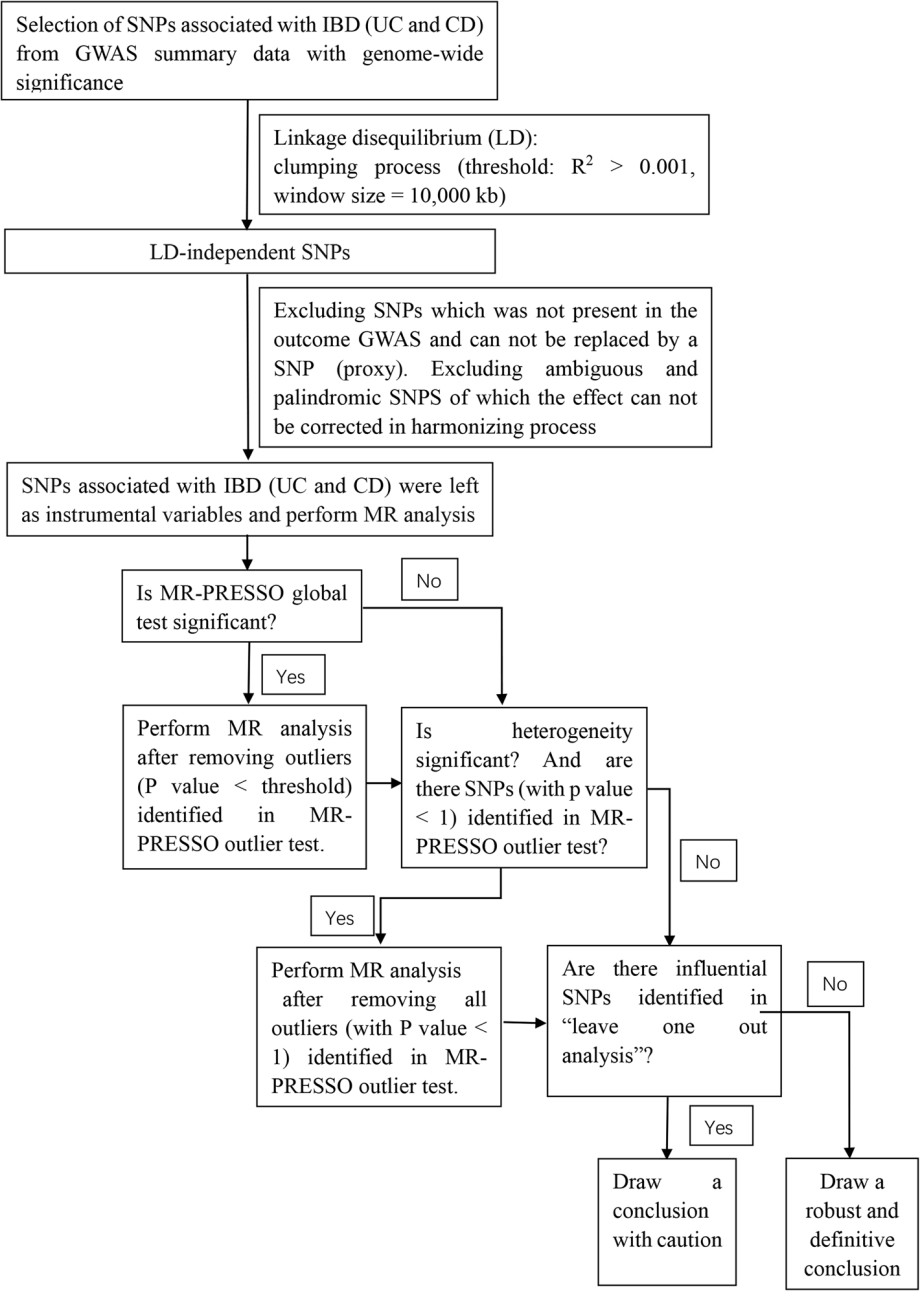
Flow chart about the analytical methods and how the MR analysis was performed step-by-step

In our study, we firstly performed MR analysis with all the above-selected SNPs as IVs. If the MR-PRESSO analysis detected a significant horizontal pleiotropy, we shall remove the outlier variants (with *P* value less than the threshold in the MR-PRESSO outlier test) and perform MR analysis again. After the MR-PRESSO outlier removal step, if the heterogeneity was still significant, we would perform MR analysis under the condition of removing all the SNPs of which the *P* value was less than 1 in the MR-PRESSO outlier test. At last, if potentially influential SNPs were identified in the “leave-one-out” sensitivity analysis, we should draw the conclusion with caution. A flow chart about the analytical methods and how the MR analysis was performed step-by-step was shown in Fig. 1.

### Ethics

Our analysis used published study or publicly available GWAS summary data. No original data was collected for this manuscript, and thus, no ethical committee approval was required. Each study included was approved by their institutional ethics review committees, and all participants provided written informed consent.

All statistical analyses were conducted using R version 3.6.3 (R Foundation for Statistical Computing, Vienna, Austria) using the Two-Sample MR package [27]. *P* values < 0.05 were considered statistically significant. In multiple testing, an adjusted *P* value after Bonferroni correction (*P* < 0.05/N, *N* = the number of testing methods) was considered statistically significant.

## Results

### Selection of instrumental variables

Detailed information of LD-independent SNPs (after clumping process) for exposure (IBD, UC, and CD) was listed in Additional file 1. The listed SNPs would be excluded in the following situations: first, in the process of extracting particular SNPs from the outcome (BMDs) GWAS, a particular requested SNP was not present in and a proxy that was in LD with the requested SNP could not be searched from the outcome GWAS. Second, the effect of ambiguous SNPs with non-concordant alleles or palindromic SNPs with ambiguous strand could not be corrected. Eventually, the number of SNPs selected as IVs for exposure in further analyses would be equal to or less than that listed in Additional file 1. *F* statistics for every instrument-exposure association were much greater than 10 in our study, demonstrating the small possibility of weak instrumental variable bias.

### Two-sample Mendelian randomization analysis for causal link of IBD with BMDs

**Fig. 2:**
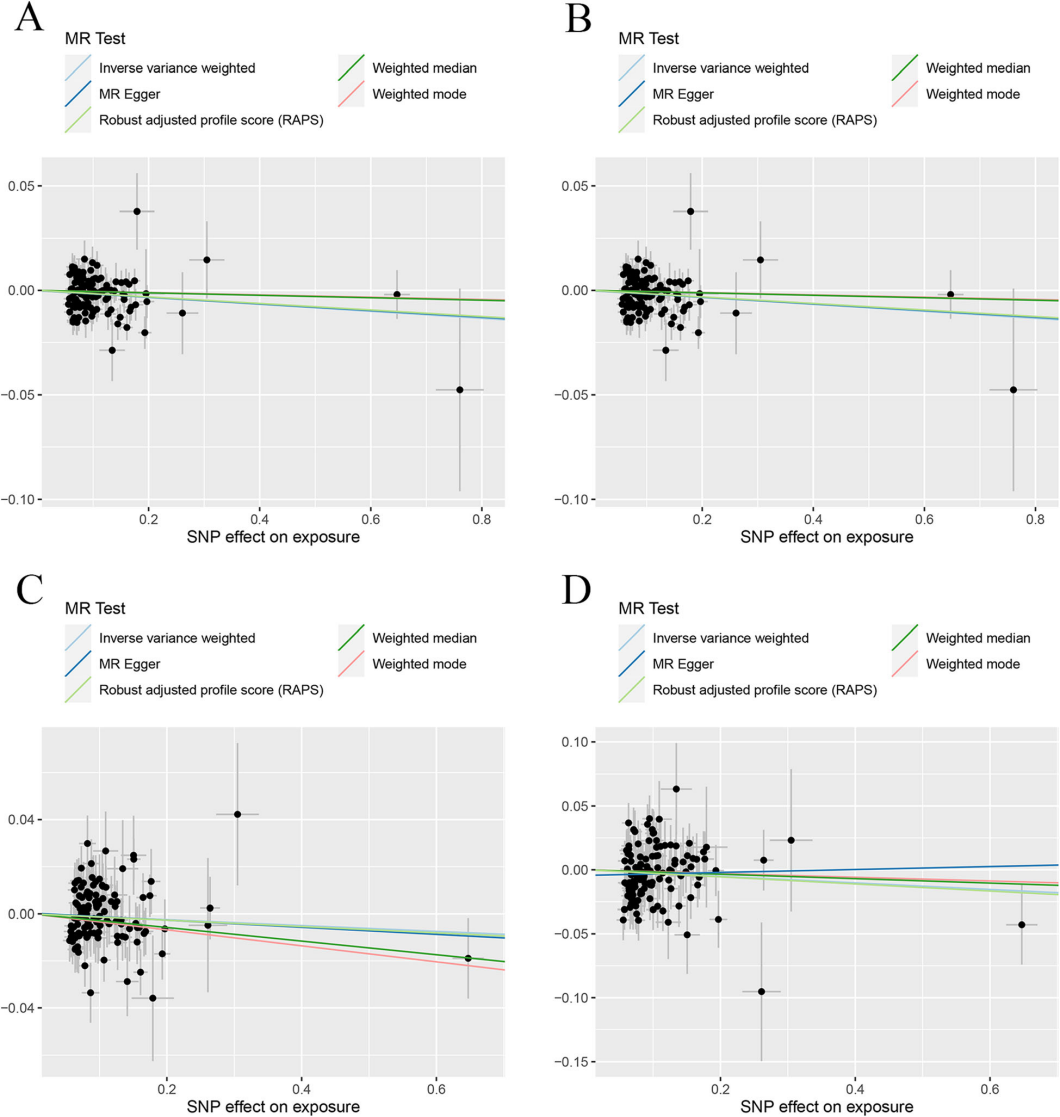
Scatter plots for MR analyses of the causal effect of IBD on BMDs in initial practice. **a** TB-BMD. **b** FN-BMD. **c** LS-BMD. **d** FA-BMD. Analyses were conducted using the conventional IVW, MBE, WMM, MR-Egger, and MR.RAPS methods. The slope of each line corresponding to the estimated MR effect per method

The MR estimates from different methods of assessing the causal effect of IBD on BMDs were presented in Table 1. MR estimates of assessing the causal effect of IBD on BMDs at different steps were presented in Additional file 2: Table S1. The results of Table 1 which contained the last step of MR estimates of Additional file 2: Table S1 demonstrated that genetically predicted IBD was negatively associated with the level of TB-BMD (IVW: *β* (95%CI) − 0.017 (− 0.029, − 0.0046), *P* = 0.0067; MR.RAPS: *β* (95%CI) − 0.016 (− 0.028, − 0.0028), *P* = 0.017) and FN-BMD (IVW: *β* (95%CI) − 0.019 (− 0.035, − 0.0032), *P* = 0.018; MBE: *β* (95%CI) − 0.049 (− 0.084, − 0.013), *P* = 0.0079; WMM: *β* (95%CI) − 0.024 (− 0.047, − 0.00035), *P* = 0.047; MR.RAPS: *β* (95%CI) − 0.018 (− 0.034, − 0.0014), *P* = 0.034) in initial practice. However, no causal effect of IBD on LS-BMD or FA-BMD was found in this part using an adjusted *P* value after Bonferroni correction (*P* < 0.01). Heterogeneity tests highlighted the existence of heterogeneity in TB-BMD (IVW, *Q* (df) 146.2 (118), *P* = 0.040; MR-Egger, *Q* (df) 146.20 (117), *P* = 0.035). Our analysis suggested no significant evidence of horizontal pleiotropy (as indicated by MR-Egger regression intercept close to zero, with a *P* value larger than 0.05). It was likely that there were SNPs exhibited horizontal pleiotropy in this part (which then tended to cancel out when the estimates were combined together in meta-analysis/Egger regression). The estimated effect sizes of the SNPs on both the exposure (IBD) and BMD outcomes are displayed in scatter plots (Fig. 2). The funnel plots providing an indication of where there existed directional horizontal pleiotropy for each outcome were shown in Additional file 3**:** Fig. S1. Plots of leave-one-out analysis which were shown in Additional file 3: Fig. S2 demonstrated that there was a potentially influential SNP driving the causal link between IBD and FN-BMD. Thus, we need to carefully interpret the result and draw a cautious conclusion.

**Table 1:** MR estimates from different methods of assessing the causal effect of IBD on BMDs

BMDs	Step^#^	No. of SNP	IVW				MBE		WMM		MR-Egger						MR.RAPS	
			*β* (95%CI)	*P* value	Cochran *Q* statistics (df)	*P* value	*β* (95%CI)	*P* value	*β* (95%CI)	*P* value	Slope (95%CI)	*P* value	Intercept (Se)	*P* value	Cochran *Q* statistics (df)	*P* value	*β* (95%CI)	*P* value
IBD and TB-BMD	3	119	− 0.017 (− 0.029, − 0.0046)	0.0067	146.2 (118)	0.040	− 0.0054 (− 0.031, 0.020)	0.67	− 0.0059 (− 0.024, 0.013)	0.53	− 0.016 (− 0.045, 0.013)	0.29	− 9.89e−5 (0.0016)	0.95	146.20 (117)	0.035	− 0.016 (− 0.028, − 0.0028)	0.017
IBD and FN-BMD	3	118	− 0.019 (− 0.035, − 0.0032)	0.018	140.57 (117)	0.068	− 0.049 (− 0.084, − 0.013)	0.0079	− 0.024 (− 0.047, − 0.00035)	0.047	− 0.0025 (− 0.039, 0.034)	0.90	− 0.0020 (0.0020)	0.33	139.42 (116)	0.068	− 0.018 (− 0.034, − 0.0014)	0.034
IBD and LS-BMD	3	120	− 0.012 (− 0.030, 0.0053)	0.17	135.03 (119)	0.15	− 0.034 (− 0.070, 0.0023)	0.069	− 0.029 (− 0.056, − 0.0018)	0.037	− 0.015 (− 0.057, 0.027)	0.48	0.00032 (0.0023)	0.89	125.00 (118)	0.14	− 0.013 (− 0.032, 0.0047)	0.15
IBD and FA-BMD	3	124	− 0.026 (− 0.057, 0.0058)	0.11	142.47 (123)	0.11	− 0.015 (− 0.084, 0.055)	0.68	− 0.017 (− 0.064, 0.029)	0.46	0.012 (− 0.063, 0.086)	0.76	− 0.0044 (0.0041)	0.29	141.14 (122)	0.11	− 0.028 (− 0.060, 0.0049)	0.095
IBD and TB-BMD(R*)	3	102	− 0.016 (− 0.027, − 5.46e−3)	0.0033	102.48 (101)	0.44	− 0.012 (− 0.037, 1.28e−2)	0.35	− 0.012 (− 0.030, 5.82e−3)	0.19	− 0.026 (− 0.052, − 9.06e−5)	0.052	0.0012 (0.0015)	0.42	101.82 (100)	0.43	− 0.016 (− 0.027, − 4.39e−3)	0.0064
IBD and FN-BMD(R*)	3	100	− 0.014 (− 0.030, 0.0014)	0.074	113.58 (99)	0.15	− 0.042 (− 0.078, − 0.0054)	0.027	− 0.028 (− 0.051, − 0.0044)	0.020	− 0.022 (− 0.058, 0.015)	0.24	0.00094 (0.0021)	0.66	113.36 (98)	0.14	− 0.015 (− 0.032, 0.0021)	0.086
IBD and LS-BMD(R*)	3	101	0.00062 (− 0.018, 0.020)	0.95	122.96 (100)	0.059	− 0.021 (− 0.056, 0.015)	0.27	− 0.022 (− 0.051, 0.0076)	0.15	− 0.030 (− 0.073, 0.013)	0.18	0.0039 (0.0025)	0.13	120.13 (99)	0.073	0.0023 (− 0.018, 0.022)	0.82
IBD and FA-BMD(R*)	3	105	− 0.026 (− 0.058, 0.0049)	0.098	116.14 (104)	0.20	− 0.012 (− 0. 082, 0.058)	0.75	− 0.011 (− 0.059, 0.037)	0.65	− 0.0017 (− 0.076, 0.073)	0.96	− 0.0031 (0.0044)	0.48	115.56 (103)	0.19	− 0.028 (− 0.062, 0.0051)	0.097

Step^#^: 3, MR analysis after removing all the SNPs (with *P* value less than 1 in MR-PRESSO outlier test); R*, in replication practice

*TB-BMD* total body bone mineral density, *FN-BMD* femoral neck bone mineral density, *LS-BMD* lumbar spine bone mineral density, *FA-BMD* forearm bone mineral density, *β* beta coefficient, *Se* standard error, *SNP* single nucleotide polymorphism, *MR* Mendelian randomization, *IVW* inverse variance weighting, *MR-PRESSO* MR-Pleiotropy RESidual Sum and Outlier method, *MBE* mode-based estimate method, *WMM* weighted median method, *MR.RAPS* robust adjusted profile score

**Fig. 3:**
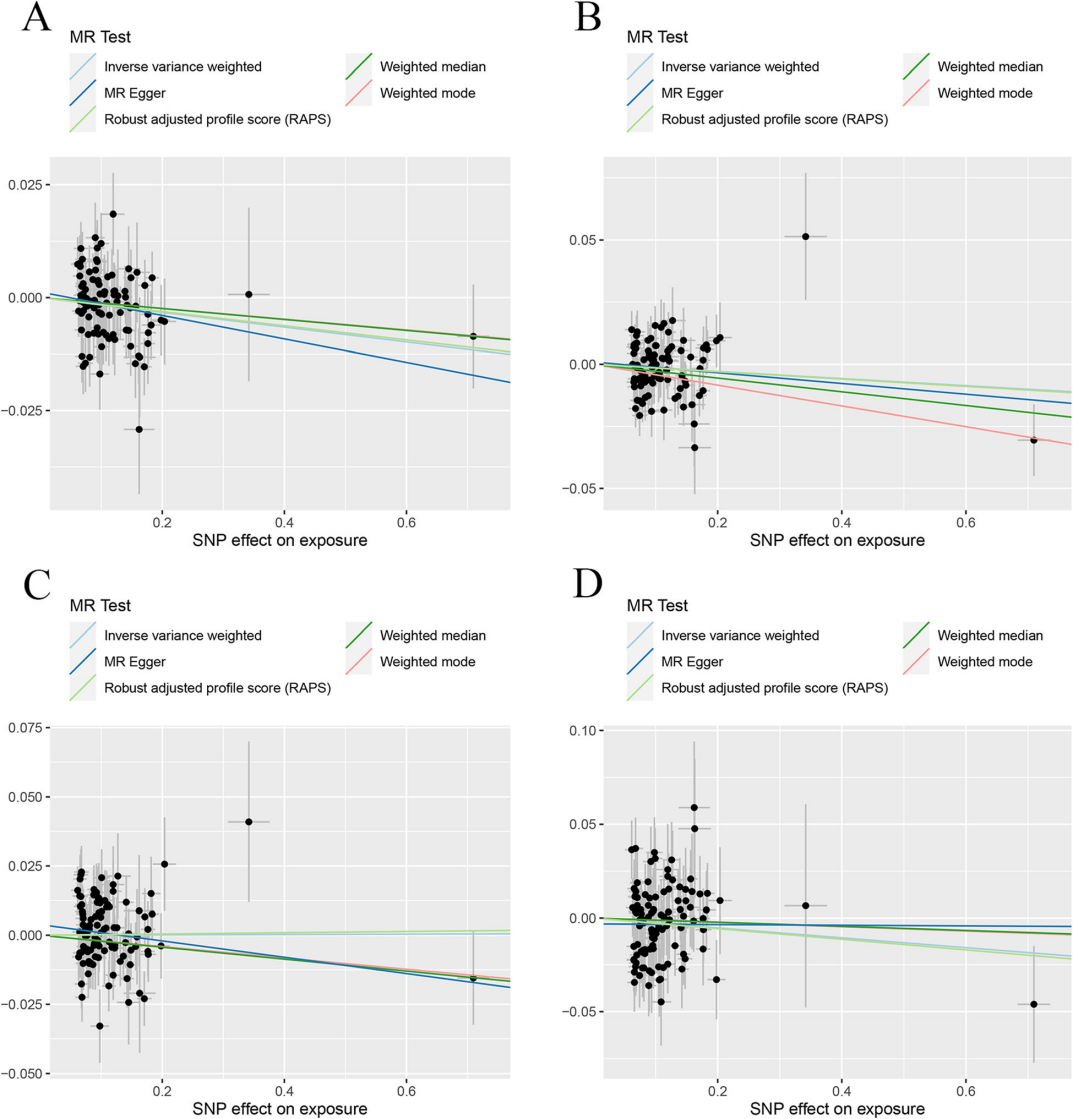
Scatter plots for MR analyses of the causal effect of IBD on BMDs in replicative practice. **a** TB-BMD. **b** FN-BMD. **c** LS-BMD. **d** FA-BMD. Analyses were conducted using the conventional IVW, MBE, WMM, MR-Egger, and MR.RAPS methods. The slope of each line corresponding to the estimated MR effect per method

In replication practice, the sample size of IBD was much larger than that in initial practice. The results of Table 1 showed the strong causal link of IBD and TB-BMD (IVW: *β* (95%CI) − 0.016 (− 0.027, − 5.46e−3), *P* = 0.0033; MR.RAPS: *β* (95%CI) − 0.016 (− 0.027, − 4.39e−3), *P* = 0.0064) and FN-BMD (MBE: *β* (95%CI) − 0.042 (− 0.078, − 0.0054), *P* = 0.027; WMM: *β* (95%CI) − 0.028 (− 0.051, − 0.0044), *P* = 0.020), which were consistent with that in initial practice. However, no causal effect of IBD on LS-BMD and FA-BMD was found in this section. We detected no heterogeneity and pleiotropy in this part. The scatter plots and funnel plots for each outcome in replication practice were shown in Fig. 3 and Additional file 3: Fig. S3. Plots of the leave-one-out analysis (Additional file 3: Fig. S4) demonstrated that the causal link between IBD and FN-BMD was driven by potentially influential SNPs, and we should carefully interpret the result and draw a cautious conclusion.

The *F* statistics for instrument IBD are 136.97 in the initial practice and 86.16 in the replication practice, demonstrating the small possibility of weak instrumental variable bias. As the results mentioned above, we may conclude the causal effect of genetically predicted IBD on TB-BMD but not on LS-BMD or FA-BMD. As to FN-BMD, the results of MR analysis in initial practice and replication practice were driven by potentially influential SNPs identified in the “leave one out” analysis, and we cannot draw a robust or definitive conclusion.

### Two-sample Mendelian randomization analysis for causal link of UC with BMDs

**Fig. 4:**
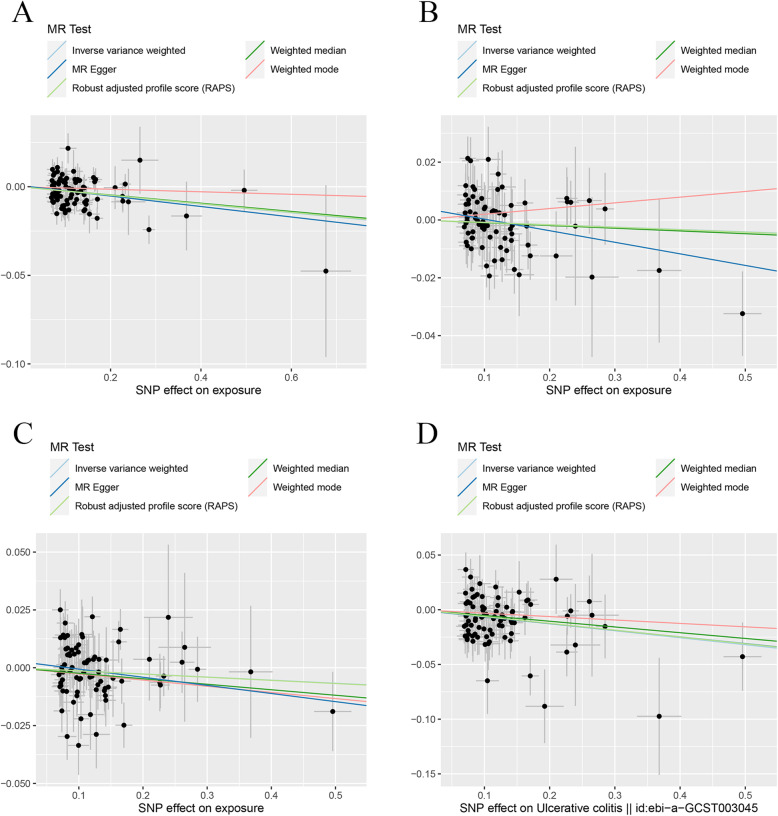
Scatter plots for MR analyses of the causal effect of UC on BMDs in initial practice. **a** TB-BMD. **b** FN-BMD. **c** LS-BMD. **d** FA-BMD. Analyses were conducted using the conventional IVW, MBE, WMM, MR-Egger, and MR.RAPS methods. The slope of each line corresponding to the estimated MR effect per method

Table 2 containing the MR estimates from different methods of assessing the causal effect of UC on BMDs demonstrated that genetically predicted UC was negatively associated with the level of TB-BMD (IVW; *β* (95%CI) − 0.024 (− 0.037, − 0.012), *P* = 0.00011; WMM: *β* (95%CI) − 0.023 (− 0.041, − 0.0053), *P* = 0.013; MR.RAPS: *β* (95%CI) − 0.024 (− 0.037, − 0.011), *P* = 0.00037) and FA-BMD (IVW: *β* (95%CI) − 0.064 (− 0.096, − 0.032), *P* = 7.79e−5; WMM: *β* (95%CI) − 0.052 (0.10, − 0.0052), *P* = 0.025; MR.RAPS: *β* (95%CI) − 0.062 (− 0.095, − 0.029), *P* = 2.22e−4). However, no causal effect of UC on FN-BMD or LS-BMD was found in this initial practice using an adjusted *P* value after Bonferroni correction (*P* < 0.01). MR estimates of assessing the causal effect of UC on BMDs at different steps were presented in Additional file 2: Table S2. MR-Egger regression tests and heterogeneity tests suggested no significant horizontal pleiotropy and heterogeneities in this part. The scatter plots, funnel plots, and “leave-one-out analysis” plots were shown in Fig. 4 and Additional file 3: Fig. S5 and Fig. S6.

**Table 2:** MR estimates from different methods of assessing the causal effect of UC on BMDs

BMDs	Step^#^	No. of SNP	IVW				MBE		WMM		MR-Egger						MR.RAPS	
			*β* (95%CI)	*P* value	Cochran *Q* statistics (df)	*P* value	*β* (95%CI)	*P* value	*β* (95%CI)	*P* value	Slope (95%CI)	*P* value	Intercept (Se)	*P* value	Cochran *Q* statistics (df)	*P* value	*β* (95%CI)	*P* value
UC and TB-BMD	2	83	− 0.024 (− 0.037, − 0.012)	0.00011	96.49 (82)	0.13	− 0.0071 (− 0.038, 0.024)	0.67	− 0.023 (− 0.041, − 0.0053)	0.013	− 0.030 (− 0.061, 0.0016)	0.067	0.00069 (0.002)	0.73	96.34 (81)	0.12	− 0.024 (− 0.037, − 0.011)	0.00037
UC and FN-BMD	2	78	− 0.0082 (− 0.025, 0.0081)	0.32	84.27 (77)	0.27	0.020 (− 0.029, 0.068)	0.45	− 0.0095 (− 0.034, 0.015)	0.43	− 0.040 (− 0.079, − 0.00065)	0.0499	0.0043 (0.0025)	0.087	81.06 (76)	0.32	− 0.0085 (− 0.025, 0.0086)	0.33
UC and LS-BMD	2	80	− 0.013 (− 0.034, 0.0069)	0.20	100.64 (79)	0.051	− 0.027 (− 0.065, 0.012)	0.18	− 0.024 (− 0.051, 0.0031)	0.083	− 0.035 (− 0.085, 0.014)	0.17	0.0029 (0.0031)	0.36	99.51 (78)	0.051	− 0.014 (− 0.034, 0.0065)	0.18
UC and FA-BMD	1	82	− 0.064 (− 0.096, − 0.032)	7.79e−5	82.65 (81)	0.43	− 0.031 (− 0.098, 0.036)	0.38	− 0.052 (0.10, − 0.0052)	0.025	− 0.061 (− 0.14, 0.019)	0.14	− 0.00038 (0.0050)	0.94	82.65 (80)	0.40	− 0.062 (− 0.095, − 0.029)	2.22e−4
UC and TB-BMD(R*)	2	72	− 0.021 (− 0.033, − 0.0086)	0.00086	83.88 (71)	0.14	− 0.021 (− 0.044, 0.0027)	0.087	− 0.023 (− 0.041, − 0.0059)	0.025	− 0.028 (− 0.058, 0.0013)	0.065	0.0011 (0.0020)	0.59	83.53 (70)	0.13	− 0.022 (− 0.035, − 0.0084)	0.0014
UC and FN-BMD(R*)	1	68	− 0.0082 (− 0.025, 0.0087)	0.34	82.25 (67)	0.099	0.0089 (− 0.028, 0.046)	0.64	0.0039 (− 0.022, 0.029)	0.77	− 0.026 (− 0.064, 0.012)	0.19	0.0026 (0.0026)	0.31	80.98 (66)	0.10	− 0.0087 (− 0.027, 0.0094)	0.35
UC and LS-BMD(R*)	3	64	− 0.0094 (− 0.027, 0.0086)	0.30	63.60 (63)	0.46	− 0.015 (− 0.049, 0.019)	0.39	− 0.016 (− 0.043, 0.012)	0.27	− 0.029 (− 0.069, 0.011)	0.16	0.0030 (0.0028)	0.29	62.45 (62)	0.46	− 0.011 (− 0.030, 0.0080)	0.26
UC and FA-BMD(R*)	1	72	− 0.044 (− 0.075, − 0.014)	0.0043	59.11 (71)	0.84	− 0.039 (− 0.10, 0.023)	0.22	− 0.043 (− 0.092, 0.0056)	0.083	− 0.076 (− 0.15, − 0.0052)	0.039	0.0047 (0.0048)	0.33	58.16 (70)	0.84	− 0.044 (− 0.076, − 0.012)	0.0074

Step^#^: 1, MR analysis with the complete selected SNPs; 2, MR analysis after removing the SNPs (with *P* value less than threshold in MR-PRESSO outlier test); 3, MR analysis after removing all the SNPs (with *P* value less than 1 in MR-PRESSO outlier test); R*, in replication practice

*TB-BMD* total body bone mineral density, *FN-BMD* femoral neck bone mineral density, *LS-BMD* lumbar spine bone mineral density, *FA-BMD* forearm bone mineral density, *β* beta coefficient, *Se* standard error, *SNP* single nucleotide polymorphism, *MR* Mendelian randomization, *IVW* inverse variance weighting, *MR-PRESSO* MR-Pleiotropy RESidual Sum and Outlier method, *MBE* mode-based estimate method, *WMM* weighted median method, *MR.RAPS* robust adjusted profile score

**Fig. 5:**
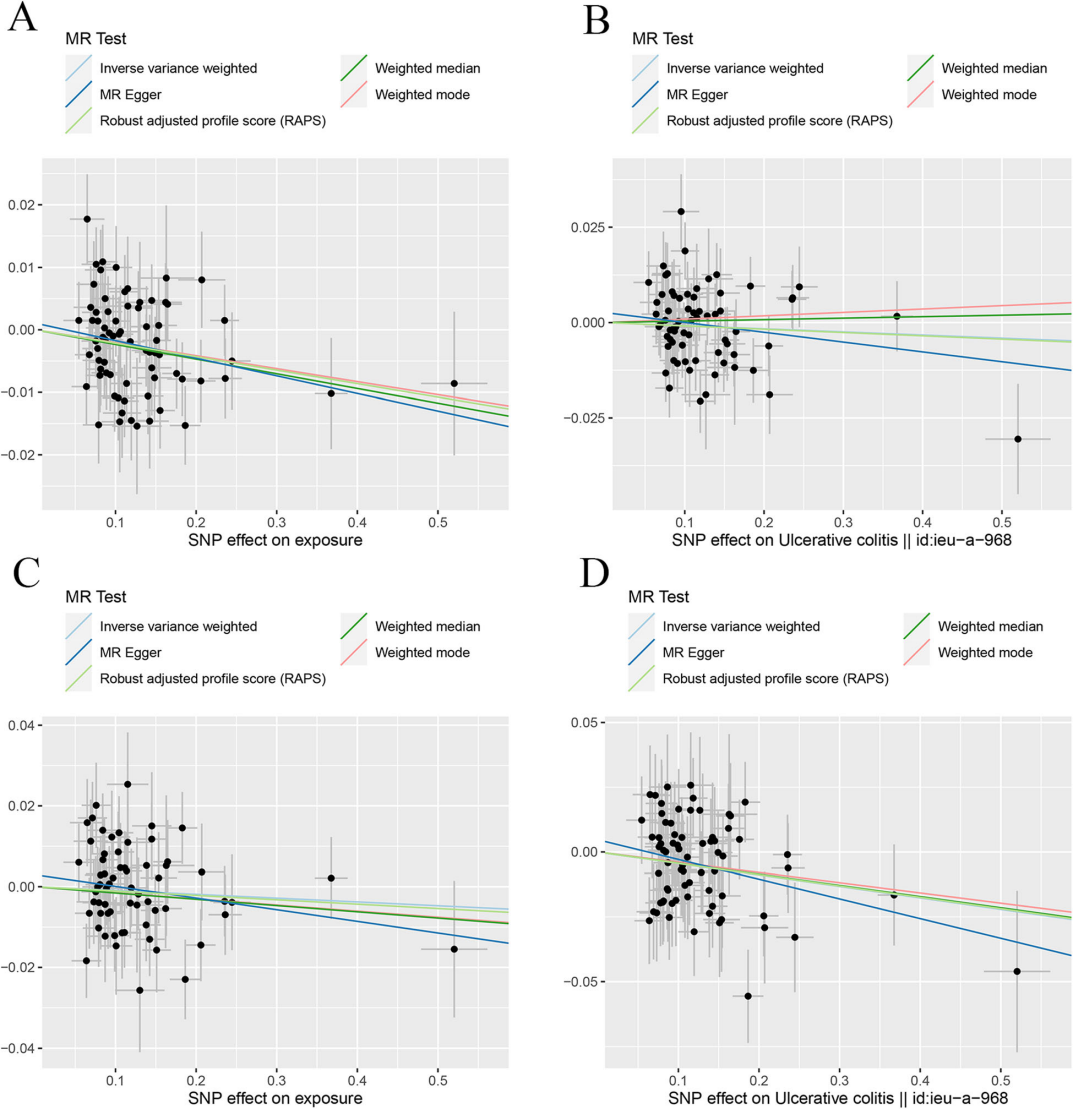
Scatter plots for MR analyses of the causal effect of UC on BMDs in replicative practice. **a** TB-BMD. **b** FN-BMD. **c** LS-BMD. **d** FA-BMD. Analyses were conducted using the conventional IVW, MBE, WMM, MR-Egger, and MR.RAPS methods. The slope of each line corresponding to the estimated MR effect per method

In the replication practice, Table 2 demonstrated the negative causal link between UC and TB-BMD (using the IVW, WMM, and MR.RAPS methods) and FA-BMD (using the IVW, MR-Egger regression, and MR.RAPS methods), which was consistent with that in initial practice. In the FN-BMD and LS-BMD groups, no causal effect of UC on decreased FN-BMD or LS-BMD was found. No directional horizontal pleiotropy and heterogeneities were detected in this section. The scatter plots and funnel plots were shown in Fig. 5 and Additional file 3: Fig. S7. Plots of the leave-one-out analysis (Additional file 3: Fig. S8) demonstrated that there was no potentially influential SNP driving the causal link and our conclusion was of stability.

The *F* statistics for instrument UC in the initial practice and the replication practice are 103.21 and 89.11, respectively. Summarizing the results of Table 2, we could receive the definite causal effect of genetically predicted UC on TB-BMD and FA-BMD but not on FN-BMD or LS-BMD.

### Two-sample Mendelian randomization analysis for causal link of CD with BMDs

**Fig. 6:**
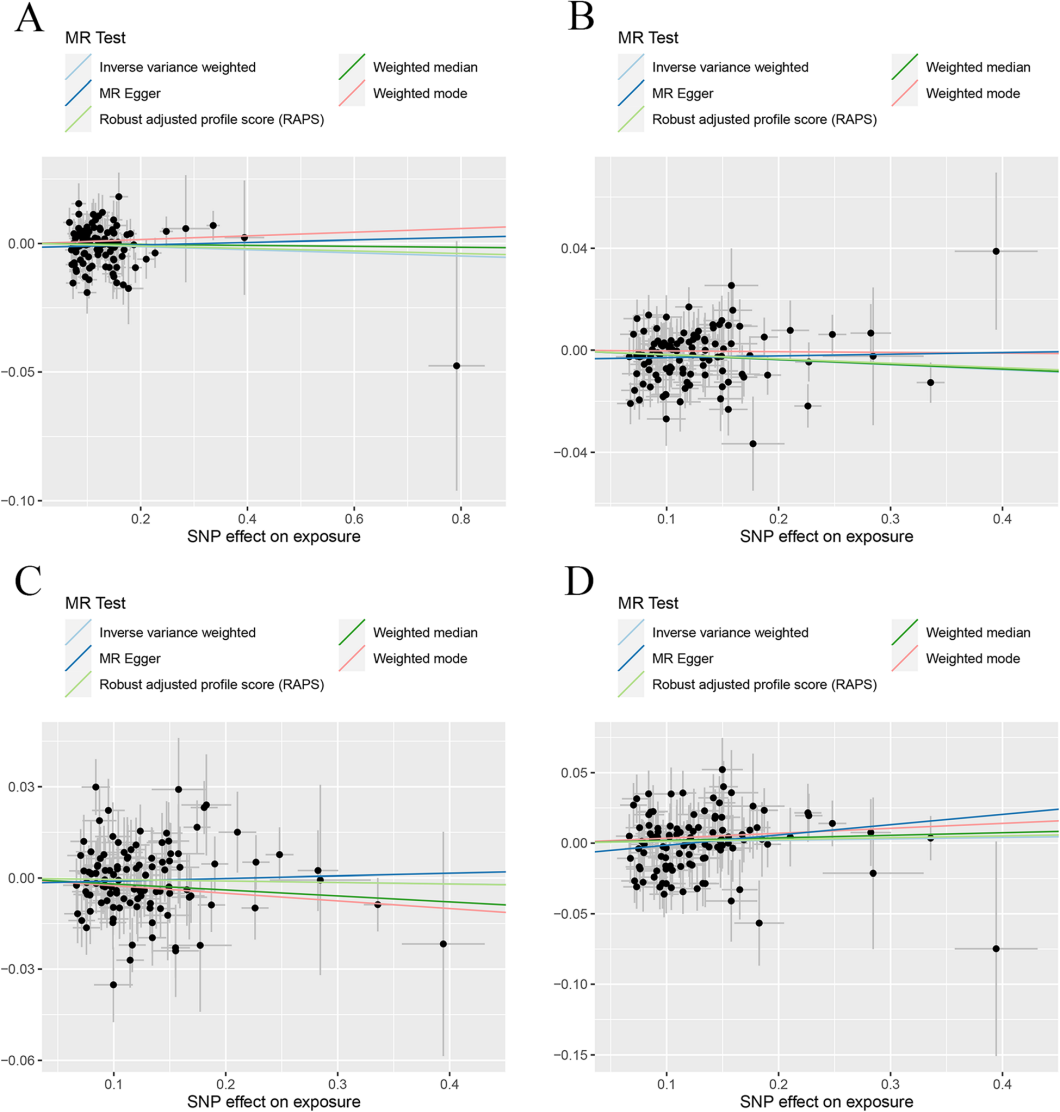
Scatter plots for MR analyses of the causal effect of CD on BMDs in initial practice. **a** TB-BMD. **b** FN-BMD. **c** LS-BMD. **d** FA-BMD. Analyses were conducted using the conventional IVW, MBE, WMM, MR-Egger, and MR.RAPS methods. The slope of each line corresponding to the estimated MR effect per method

In the two-sample MR analysis, we found CD did not have a causal link with the change of TB-BMD, LS-BMD, and FA-BMD under different MR methods (Table 3). MR estimates of assessing the causal effect of CD on BMDs at different steps were presented in Additional file 2: Table S3. As to FN-BMD, a negative causal link was found using the IVW method (*β* (95%CI) − 0.019 (− 0.034, − 0.0050), *P* = 0.0083) and the MR.RAPS method (*β* (95%CI) − 0.017 (− 0.032, − 0.0019), *P* = 0.027). No significant evidence of horizontal pleiotropy and heterogeneities were detected in this section. The scatter plots and funnel plots were shown in Fig. 6 and Additional file 3: Fig. S9. The plots of the leave-one-out analysis (Additional file 3: Fig. S10) demonstrated no potentially influential SNPs driving the causal link between CD and BMDs.

**Table 3:** MR estimates from different methods of assessing the causal effect of CD on BMDs

BMDs	Step^#^	No. of SNP	IVW				MBE		WMM		MR-Egger						MR.RAPS	
			*β* (95%CI)	*P* value	Cochran *Q* statistics (df)	*P* value	*β* (95%CI)	*P* value	*β* (95%CI)	*P* value	Slope (95%CI)	*P* value	Intercept (Se)	*P* value	Cochran *Q* statistics (df)	*P* value	*β* (95%CI)	*P* value
CD and TB-BMD	3	103	− 0.0061 (− 0.016, 0.0040)	0.24	107.79 (102)	0.33	0.0071 (− 0.016, 0.031)	0.55	− 0.0018 (− 0.017, 0.013)	0.81	0.0047 (− 0.024, 0.033)	0.75	− 0.0015 (0.0019)	0.43	107.12 (101)	0.32	− 0.0049 (− 0.015, 0.0056)	0.36
CD and FN-BMD	3	104	− 0.019 (− 0.034, − 0.0050)	0.0083	127.33 (103)	0.052	− 0.0030 (− 0.036, 0.030)	0.86	− 0.019 (− 0.039, 0.0024)	0.082	0.0066 (− 0.033, 0.046)	0.74	− 0.0036 (0.0026)	0.17	125.00 (102)	0.061	− 0.017 (− 0.032, − 0.0019)	0.027
CD and LS-BMD	2	107	− 0.0048 (− 0.021, 0.011)	0.55	125.92 (106)	0.091	− 0.025 (− 0.064, 0.014)	0.21	− 0.020 (− 0.042, 0.0030)	0.089	0.0084 (− 0.036, 0.053)	0.71	− 0.0018 (0.0030)	0.53	125.45 (105)	0.085	− 0.0049 (− 0.021, 0.011)	0.54
CD and FA-BMD	3	108	0.010 (− 0.018)	0.48	123.69 (107)	0.13	0.035 (− 0.022, 0.092)	0.23	0.018 (− 0.023, 0.060)	0.39	0.073 (− 0.0039, 0.15)	0.066	− 0.0088 (0.0051)	0.088	120.33 (106)	0.16	0.013 (− 0.016, 0.042)	0.38
CD and TB-BMD(R*)	3	92	− 0.0072 (− 0.018, 0.0032)	0.17	103.94 (91)	0.17	− 0.0082 (− 0.028, 0.012)	0.43	− 0.0096 (− 0.027, 0.0082)	0.29	− 0.012 (− 0.037, 0.012)	0.32	0.00080 (0.0017)	0.64	103.69 (90)	0.15	− 0.0070 (− 0.018, 0.0037)	0.20
CD and FN-BMD(R*)	3	90	− 0.022 (− 0.036, − 0.0072)	0.0034	111.59 (89)	0.053	− 0.033 (− 0.061, − 0.0062)	0.018	− 0.028 (− 0.051, − 0.0049)	0.018	− 0.015 (− 0.047, 0.018)	0.39	− 0.0011 (0.0023)	0.63	111.30 (88)	0.047	− 0.023 (− 0.038, − 0.0073)	0.0039
CD and LS-BMD(R*)	3	84	− 0.0022 (− 0.018, 0.013)	0.78	78.79 (83)	0.61	− 0.018 (− 0.051, 0.015)	0.28	− 0.015 (− 0.041, 0.010)	0.24	0.0024 (− 0.032, 0.036)	0.89	− 0.00071 (0.0024)	0.77	78.71 (82)	0.58	− 0.0017 (− 0.018, 0.014)	0.84
CD and FA-BMD(R*)	3	95	− 0.0095 (− 0.036, 0.017)	0.49	98.95 (94)	0.34	0.0012 (− 0.056, 0.058)	0.97	0.00063 (− 0.039, 0.041)	0.98	0.0062 (− 0.056, 0.069)	0.85	− 0.0024 (0.0044)	0.59	98.64 (93)	0.32	− 0.0066 (− 0.035, 0.021)	0.64

Step^#^: 2, MR analysis after removing the SNPs (with *P* value less than threshold in MR-PRESSO outlier test); 3, MR analysis after removing all the SNPs (with *P* value less than 1 in MR-PRESSO outlier test); R*, in replication practice

*TB-BMD* total body bone mineral density, *FN-BMD* femoral neck bone mineral density, *LS-BMD* lumbar spine bone mineral density, *FA-BMD* forearm bone mineral density, *β* beta coefficient, *Se* standard error, *SNP* single nucleotide polymorphism, *MR* Mendelian randomization, *IVW* inverse variance weighting, *MR-PRESSO* MR-Pleiotropy RESidual Sum and Outlier method, *MBE* mode-based estimate method, *WMM* weighted median method, *MR.RAPS* robust adjusted profile score

**Fig. 7:**
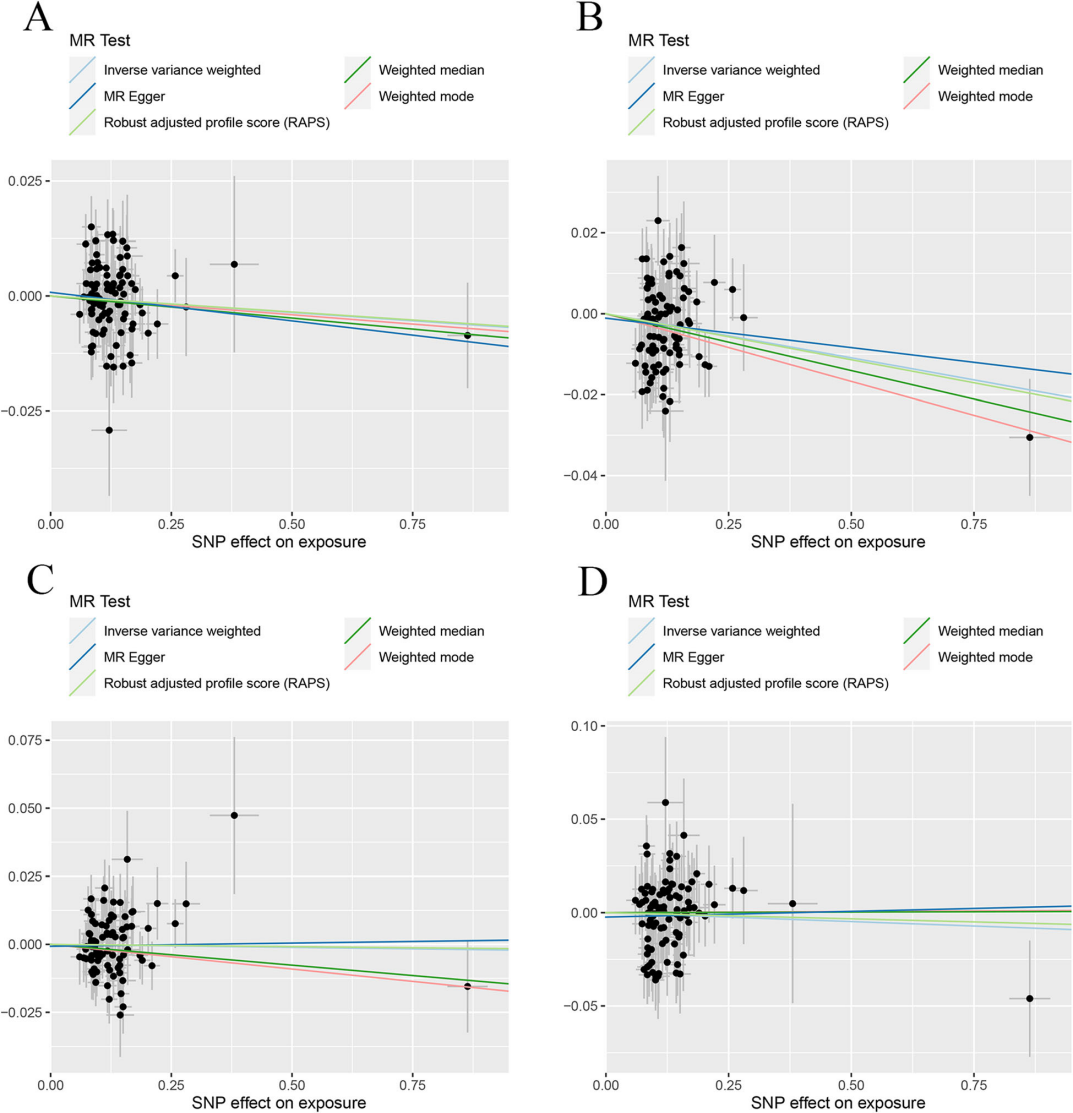
Scatter plots for MR analyses of the causal effect of CD on BMDs in replicative practice. **a** TB-BMD. **b** FN-BMD. **c** LS-BMD. **d** FA-BMD. Analyses were conducted using the conventional IVW, MBE, WMM, MR-Egger, and MR.RAPS methods. The slope of each line corresponding to the estimated MR effect per method

In the replication practice, a negative causal link was found between CD and FN-BMD using the IVW method (*β* (95%CI) − 0.022 (− 0.036, − 0.0072), *P* = 0.0034), MBE method (*β* (95%CI) − 0.033 (− 0.061, − 0.0062), *P* = 0.018), WMM (*β* (95%CI) − 0.028 (− 0.051, − 0.0049), *P* = 0.018), and MR.RAPS method (*β* (95%CI) − 0.023 (− 0.038, − 0.0073), *P* = 0.0039). No significantly causal link between CD and the change of TB-BMD, LS-BMD, or FA-BMD was found under the different MR methods. Heterogeneity tests demonstrated no existence of significant heterogeneity except for FN-BMD (MR-Egger Cochran statistics (df) 111.30 (88), *P* = 0.047). And no directional horizontal pleiotropy was detected by MR-Egger tests. The scatter plots and funnel plots were shown in Fig. 7 and Additional file 3: Fig. S11. The plots of the leave-one-out analysis (Additional file 3: Fig. S12) demonstrated no potentially influential SNPs driving the causal link between CD and BMDs in the replication practice.

The *F* statistics for instrument CD in the initial practice and replication practice are 160.18 and 102.38, respectively. Summarizing the results above, we could conclude there was a causal link of CD on FN-BMD, but not on TB-BMD, LS-BMD, or FA-BMD.

## Discussion

In this study, we used summary statistics from GWASs to identify the causal relationships between IBD (including UC and CD) and BMD at different skeletal sites. The results suggested that UC causally decreased TB-BMD and FA-BMD, the estimated effect sizes of UC on FN-BMD were not significant with an adjusted *P* value after Bonferroni correction, and UC did not definitely decrease LS-BMD, implying that the causal effects of UC on BMD at different skeletal sites were different. Previous studies suggested that only cortical thickness and cortical BMD were different, with smaller values in the UC patients than controls, and no differences were found in the trabecular or endocortical compartments [34]. The adult human skeleton is composed of 80% cortical bone and 20% trabecular bone overall. The vertebra is composed of the cortical to trabecular bone in a ratio of 25:75; this ratio is 50:50 in the femoral head and 95:5 in the radial diaphysis [35]. Thus, we inferred the different effect of UC on BMDs at different skeletal sites may be differently associated with various components of the bone, since the bone from different skeletal sites differs in composition (e.g., different proportions of the trabecular and cortical bones). Radial diaphysis and total body have the highest percentage of the cortical bone in the skeletal sites studied here. From our results, we found that the genetically predicted UC significantly caused a decrease in FA-BMD and TB-BMD. The femoral head and the vertebra have the lowest percentage of the cortical bone for the BMD phenotypes, and the effect of UC on FN-BMD and LS-BMD was not obvious.

Some publications reported that BMD was reduced in patients with CD but not in patients with UC [18, 19]. Haschka et al.’s research demonstrated that CD patients exhibited a more severe bone loss phenotype compared with UC patients [34]. The possible reasons might be as follows: CD is a systemic disease with a long premorbid phase, while UC is a mucosal disease with an acute onset and is often limited to the distal colonic tracts. In addition, CD has important immunological differences when compared to UC. The localization of CD is in the small intestine, and intestinal resection may cause malnutrition and estrogen deficiency [36]. However, in Schoon et al.’s research, it concluded no significant differences in BMD between patients with either CD or UC [37]. In this two-sample MR analysis assessing the causal link of IBD (including UC and CD) on BMDs, we determined a causal effect of genetically predicted UC on TB-BMD and FA-BMD, but only get a causal effect of CD on BMD, which was somewhat inconsistent with many published observational researches. The reasons for the difference between our MR analysis results and most other observational researches may be explained as follows: firstly, the results of epidemiological observational studies were affected by other related factors. For example, Bernstein et al.’s publication revealed that decreased BMD in IBD patients was related to corticosteroid use but not the disease itself [16]. The results of Andreassen et al.’s research with 113 CD patients and 113 healthy subjects, individually matched for gender, age, and body weight, showed that BMD of patients with CD was not different from that of healthy controls except for a decreased BMD of the hip in female patients, and gender, age, and body weight are the major determinants of BMD in patients with CD [38]. And in this MR analysis, the corresponding effect estimate of SNP on IBD (including UC and CD) and BMD had been adjusted for many principal components. Secondly, the results of our MR analysis might be biased by pleiotropy. We did not search through the Ensembl Project or PhenoScanner database as previous studies to screen genetic variants which are associated with confounding factors [39, 40]. We just performed the MR-PRESSO outlier test to identify and remove outlier variants. However, we deemed the possibility that pleiotropy significantly biased the results of our analysis was tiny, as several robust methods for MR have been performed, which can provide reliable inferences when some genetic variants violate the IV assumptions. Otherwise, we included an IBD (including UC and CD) GWAS dataset for replication purposes. It would make our conclusions more robust and reliable. Further MR analysis with more CD patients or more advanced methods to get less biased estimates and better precision is warranted in the future to confirm the relationship between CD and the level of BMDs. The causal effect of IBD on TB-BMD was significant and robust but not on FA-BMD or LS-BMD after Bonferroni correction. As to FN-BMD, the causal effect was the lack of stability. This might result from the cumulative effect of UC and CD on BMDs.

There are two types of pleiotropy (vertical pleiotropy and horizontal pleiotropy). Vertical pleiotropy occurs when a variant is directly associated with the exposure and another phenotype on the same biological pathway. This does not lead to the violation of the IV assumptions providing the only causal pathway from the genetic variant to the outcome passes via the exposure. Horizontal pleiotropy occurs when the second phenotype is on a different biological pathway, and so, there may exist different causal pathways from the variant to the outcome. This would violate the IV3 assumption [41]. To solve the problems that arise due to horizontal pleiotropy, several robust MR methods besides IVW have been performed in our study. The methods can be divided into three categories: consensus methods (e.g., weighted median, mode-based method), outlier-robust methods (e.g., MR-PRESSO), and modeling methods (e.g., MR-Egger and MR-RAPS), and the methods mentioned each possesses its own advantages [41]. Investigators should perform a range of robust methods that come from different categories and that operate in different ways and rely on different assumptions for valid inferences to assess the reliability of MR analyses. The other measures that might be taken to reduce the effect of horizontal pleiotropy were searching through the Ensembl Project or PhenoScanner database to identify and exclude genetic variants relating to confounding factors. We have not admitted this measure as it would not necessarily differentiate between horizontal and vertical pleiotropy, where only the former would bias MR studies. On the other hand, the exact biological function of many genetic variants is unknown.

Our research was the first MR analysis of this topic. In this study, we selected SNPs with genome-wide association and independent inheritance without any LD as IVs to detect the causal link between IBD (including UC and CD) and BMDs. To make our conclusions more robust and reliable, the outlier variants identified by the MR-PRESSO outlier test were removed step-by-step. We also utilized several robust analytical methods based on different assumptions of two-sample MR analysis with four groups of outcome summary GWAS data (TB-BMD, FN-BMD, LS-BMD, and FA-BMD) and two groups of exposure summary GWAS data. Instead of using just a few strong SNPs as IVs, we utilized many (potentially hundreds of) stringently selected weak SNPs as the IVs for our two-sample MR analysis, which usually substantially decreases the variance of the estimator. Since we included many weak instrumental variables in the analysis, the *F* statistic was used to assess the strength of the association between the genetic variants and exposure. The *F* statistics were much greater than 10 in our analysis, hinting the small possibility of weak instrumental variable bias [25]. We also carried out the MR.RAPS method, which can give a robust inference for our MR analysis with many weak IVs. Lastly, the summary GWAS data we drew for IBD (including UC and CD) and BMDs consisted uniquely of individuals of European descent and had been adjusted for many principal components, which would reduce potential bias.

Some limitations of our MR analysis need to be considered. First, the exposure and outcome studies used in two-sample MR analysis should not involve overlapping participants. We were not able to estimate the degree of overlap in the study. However, bias from sample overlap can be minimized by using strong instruments (e.g., *F* statistic much greater than 10), [42]. Second, the summary GWAS data merely concern individuals of European descent, and our results may not be fully representative of the whole population. So, we should carefully utilize our conclusion in racially and ethnically diverse populations. Third, we cannot expel the possibility that horizontal pleiotropy affected our results, even though we took steps to identify and exclude outlier variants. Fourth, each method we utilized in the analysis has its own strengths and weaknesses. However, the use of so many methods based on different assumptions may increase the possibility of getting inconsistent or contrary results and make the conclusion become obscured.

## Conclusion

In this study, our aim is to assess the causal effect of IBD (including UC and CD) on decreased BMD by using two-sample MR analysis. The results of our research got a definitively causal effect of genetically predicted UC on TB-BMD and FA-BMD but not on FN-BMD or LS-BMD, and we merely determined a causal effect of CD on FN-BMD, which was somewhat inconsistent with many published observational researches. Updated MR analysis is warranted to confirm our findings when a more advanced method to get less biased estimates and better precision or GWAS summary data with more UC and CD patients was available. Foremost, our research reminded clinicians that measures and concerted efforts for prevention of bone loss and early intervention of osteoporosis should be considered when IBD patients are diagnosed.

## Supplementary information

**Additional file 1** Detailed information of LD-independent SNPs (after clumping process) for exposure (IBD, UC and CD).

**Additional file 2:** **Table S1.** MR estimates from different methods of assessing the causal effect of IBD on BMDs step by step. **Table S2.** MR estimates from different methods of assessing the causal effect of UC on BMDs step by step. **Table S3.** MR estimates from different methods of assessing the causal effect of CD on BMDs step by step.

**Additional file 3:** **Figure S1.** Funnel plots for MR analyses of the causal effect of IBD on BMDs in initial practice (A) TB-BMD (B) FN-BMD (C) LS-BMD (D) FA-BMD. **Figure S2.** Plots of “leave-one-out” analyses for MR analyses of the causal effect of IBD on BMDs in initial practice (A) TB-BMD (B) FN-BMD (C) LS-BMD (D) FA-BMD. **Figure S3.** Funnel plots for MR analyses of the causal effect of IBD on BMDs in replicative practice (A) TB-BMD (B) FN-BMD (C) LS-BMD (D) FA-BMD. **Figure S4.** Plots of “leave-one-out” analyses for MR analyses of the causal effect of IBD on BMDs in replicative practice (A) TB-BMD (B) FN-BMD (C) LS-BMD (D) FA-BMD. **Figure S5.** Funnel plots for MR analyses of the causal effect of UC on BMDs in initial practice (A) TB-BMD (B) FN-BMD (C) LS-BMD (D) FA-BMD. **Figure S6.** Plots of “leave-one-out” analyses for MR analyses of the causal effect of UC on BMDs in initial practice (A) TB-BMD (B) FN-BMD (C) LS-BMD (D) FA-BMD. **Figure S7.** Funnel plots for MR analyses of the causal effect of UC on BMDs in replicative practice (A) TB-BMD (B) FN-BMD (C) LS-BMD (D) FA-BMD. **Figure S8.** Plots of “leave-one-out” analyses for MR analyses of the causal effect of UC on BMDs in replicative practice (A) TB-BMD (B) FN-BMD (C) LS-BMD (D) FA-BMD. **Figure S9.** Funnel plots for MR analyses of the causal effect of CD on BMDs in initial practice (A) TB-BMD (B) FN-BMD (C) LS-BMD (D) FA-BMD. **Figure S10.** Plots of “leave-one-out” analyses for MR analyses of the causal effect of CD on BMDs in initial practice (A) TB-BMD (B) FN-BMD (C) LS-BMD (D) FA-BMD. **Figure S11.** Funnel plots for MR analyses of the causal effect of CD on BMDs in replicative practice (A) TB-BMD (B) FN-BMD (C) LS-BMD (D) FA-BMD. **Figure S12.** Plots of “leave-one-out” analyses for MR analyses of the causal effect of CD on BMDs in replicative practice (A) TB-BMD (B) FN-BMD (C) LS-BMD (D) FA-BMD.

## Data Availability

The datasets supporting the conclusions of this article are available in the [repository name] repository. The GWAS summary statistics for BMDs is available in the GEnetic Factors for OSteoporosis Consortium website (GEFOS: http://www.gefos.org/) or https://www.ebi.ac.uk/gwas/downloads/summary-statistics. The GWAS summary statistics for IBD (including UC and CD) is available on the websites https://www.ibdgenetics.org/downloads.html and https://gwas.mrcieu.ac.uk/datasets/. The other data generated or analyzed during this study are available in this published article and its supplementary information files.

## Abbreviations

BMD: Bone mineral density
BMI: Body mass index
CD: Crohn’s disease
DXA: Dual-energy X-ray absorptiometry
FA-BMD: Forearm bone mineral density
FN-BMD: Femoral neck bone mineral density
GEFOS: GEnetic Factors for OSteoporosis Consortium
GWAS: Genome-wide association study
IBD: Inflammatory bowel disease
InSIDE: Instrument Strength Independent of Direct Effect
IV: Instrument variable
IVW: Inverse variance weighting
LD: Linkage disequilibrium
LS-BMD: Lumbar Spine bone mineral density
MAF: Minor allele frequency
MBE: Mode-based estimate method
MR: Mendelian randomization
MR.RAPS: Robust adjusted profile score
MR-PRESSO: MR-Pleiotropy RESidual Sum and Outlier method
RCT: Randomized controlled trial
SNP: Single-nucleotide polymorphism
TB-BMD: Total body bone mineral density
UC: Ulcerative colitis
WMM: Weighted median method
