# Supplementary material for: Mendelian randomization study of inflammatory bowel disease and bone mineral density
Source: BMC Med. 2020 Nov 10;18:312. doi: 10.1186/s12916-020-01778-5 (PMC7654011; doi:10.1186/s12916-020-01778-5)
Supplement: Supplementary file 2 — Additional file 2: Table S1. MR estimates from different methods of assessing the causal effect of IBD on BMDs step by step. Table S2. MR estimates from different methods of assessing the causal effect of UC on BMDs step by step. Table S3. MR estimates from different methods of assessing the causal effect of CD on BMDs step by step. [file 12916_2020_1778_MOESM2_ESM.zip › Additional File2 Table S1R2.docx]

Supplementary Table 1: MR estimates from different methods of assessing the causal effect of IBD on BMDs step by step

| BMDs | Step^#^ | No. of SNP | IVW | | | | Mr-presso | | MBE | | WMM | | MR-Egger | | | | | | MR.RAPS | |  |  |
| --- | --- | --- | --- | --- | --- | --- | --- | --- | --- | --- | --- | --- | --- | --- | --- | --- | --- | --- | --- | --- | --- | --- |
|  |  |  | β(95%CI) | P value | Cochran Q statistics (df) | P value | β(Se) | P value | β(95%CI ) | P value | β(95%CI ) | P value | Slope(95%CI ) | P value | Intercept(Se) | P value | Cochran Q statistics (df) | P value | β(95%CI ) | P value | | |
| IBD and TB-BMD | 1 | 128 | -0.018 ( -0.032, -0.0030) | 0.018 | 247.49 (127) | 8.78e-10 | -0.016 (0.0067) | 0.020 | -0.0059 ( -0.032, 0.020) | 0.66 | -0.0049 ( -0.023, 0.013) | 0.59 | 0.0056 ( -0.029, 0.040) | 0.75 | -0.0028 (0.0019) | 0.15 | 247.49 (126) | 8.78e-10 | -0.015 ( -0.030, -0.00086) | 0.038 | | |
|  | 2 | 125 | -0.016 (-0.029, -0.0027) | 0.018 | 193.07 (124) | 7.10e-5 | NA | NA | -0.0062 (-0.032,0.019) | 0.63 | -0.0049 (-0.023,0.013) | 0.36 | -0.0099 (0.016) | 0.59 | -0.0019 (0.0017) | 0.28 | 191.22 (123) | 7.98e-5 | -0.015 (-0.028, -0.0011) | 0.035 | | |
|  | 3 | 119 | -0.017 (-0.029, -0.0046) | 0.0067 | 146.2 (118) | 0.040 | NA | NA | -0.0054 (-0.031,0.020) | 0.67 | -0.0059 (-0.024, 0.013) | 0.53 | -0.016 (-0.045, 0.013) | 0.29 | -9.89e-5 (0.0016) | 0.95 | 146.20 (117) | 0.035 | -0.016 (-0.028, -0.0028) | 0.017 | | |
| IBD and FN-BMD | 1 | 122 | -0.012 (-0.029,0.0050) | 0.17 | 178.55 (121) | 0.00052 | -0.014 (0.0084) | 0.090 | -0.047 (-0.085, -0.010) | 0.014 | -0.022 (-0.046, 0.0017) | 0.068 | -0.0020 (-0.042, 0.038) | 0.92 | -0.0012 (0.0022) | 0.59 | 178.11 (120) | 0.00046 | -0.014 (-0.031,0.0038) | 0.13 | | |
|  | 2 | 121 | -0.014 (-0.031,0.0021) | 0.087 | 161.94 (120) | 0.0065 | NA | NA | -0.047 (-0.085, -0.010) | 0.014 | -0.023 (-0.046, 0.00028) | 0.053 | -0.0062 (-0.045, 0.033) | 0.75 | -0.00098 (0.0021) | 0.65 | 161.66 (119) | 0.0057 | -0.015 (-0.032, 0.0022) | 0.088 | | |
|  | 3 | 118 | -0.019 (-0.035, -0.0032) | 0.018 | 140.57 (117) | 0.068 | NA | NA | -0.049 (-0.084, -0.013) | 0.0079 | -0.024 (-0.047, -0.00035) | 0.047 | -0.0025 (-0.039, 0.034) | 0.90 | -0.0020 (0.0020) | 0.33 | 139.42 (116) | 0.068 | -0.018 (-0.034, -0.0014) | 0.034 | | |
| IBD and LS-BMD | 1 | 122 | -0.013 ( -0.031, 0.0058) | 0.18 | 151.65 (121) | 0.031 | NA | NA | -0.034 ( -0.070, 0.0017) | 0.064 | -0.029 ( -0.058, 0.00046) | 0.054 | -0.017 ( -0.061, 0.027) | 0.45 | 0.00051 (0.0024) | 0.83 | 151.59 (120) | 0.027 | -0.013 ( -0.032, 0.0054) | 0.16 | | |
|  | 3 | 120 | -0.012 ( -0.030, 0.0053) | 0.17 | 135.03 (119) | 0.15 | NA | NA | -0.034 ( -0.070, 0.0023) | 0.069 | -0.029 ( -0.056, -0.0018) | 0.037 | -0.015 ( -0.057, 0.027) | 0.48 | 0.00032 (0.0023) | 0.89 | 125.00 (118) | 0.14 | -0.013 ( -0.032, 0.0047) | 0.15 | | |
| IBD and FA-BMD | 1 | 126 | -0.028 (-0.062,0.0053) | 0.099 | 166.55 (125) | 0.0077 | -0.032 (0.016) | 0.054 | -0.015 (-0.080,0.051) | 0.66 | -0.019 (-0.066,0.029) | 0.44 | -0.0075 (-0.087,0.072) | 0.85 | -0.0024 (0.0044) | 0.58 | 166.14 (124) | 0.0069 | -0.030 (-0.063,0.0040) | 0.084 | | |
|  | 2 | 125 | -0.032 (-0.064,0.00025) | 0.052 | 152.21 (124) | 0.043 | NA | NA | -0.015 (-0.085,0.056) | 0.69 | -0.020 (-0.067,0.028) | 0.42 | -0.0027 (-0.079,0.074) | 0.94 | -0.0035 (0.0042) | 0.41 | 151.38 (123) | 0.042 | -0.031 (-0.064,0.0014) | 0.061 | | |
|  | 3 | 124 | -0.026 (-0.057,0.0058  ) | 0.11 | 142.47 (123) | 0.11 | NA | NA | -0.015 (-0.084,0.055) | 0.68 | -0.017 (-0.064,0.029) | 0.46 | 0.012 (-0.063,0.086) | 0.76 | -0.0044 (0.0041) | 0.29 | 141.14 (122) | 0.11 | -0.028 (-0.060,0.0049) | 0.095 | | |
| IBD and TB-BMD(R*) | 1 | 110 | -0.018 ( -0.032, -0.0038) | 0.012 | 189.43 (109) | 2.87e-6 | -0.016 (0.0062) | 0.010 | -0.012 ( -0.036, 0.012) | 0.33 | -0.012 ( -0.031, 0.0064) | 0.20 | -0.023 ( -0.057, 0.010) | 0.18 | 0.00068 (0.0020) | 0.73 | 189.22 (108) | 2.24e-6 | -0.017 ( -0.030, -0.0037) | 0.012 | | |
|  | 2 | 107 | -0.016 ( -0.028, -0.0040) | 0.0093 | 139.74 (106) | 0.016 | NA | NA | -0.012 ( -0.033, 0.0090) | 0.27 | -0.012 ( -0.030, 0.0060) | 0.19 | -0.031 ( -0.060, -0.0017) | 0.040 | 0.0019 (0.0017) | 0.27 | 138.17 (105) | 0.017 | -0.016 ( -0.028, -0.003) | 0.010 | | |
|  | 3 | 102 | -0.016 ( -0.027, -5.46e-3) | 0.0033 | 102.48 (101) | 0.44 | NA | NA | -0.012 ( -0.037, 1.28e-2) | 0.35 | -0.012 ( -0.030, 5.82e-3) | 0.19 | -0.026 ( -0.052, -9.06e-5) | 0.052 | 0.0012 (0.0015) | 0.42 | 101.82 (100) | 0.43 | -0.016 ( -0.027, -4.39e-3) | 0.0064 | | |
| IBD and FN-BMD(R*) | 1 | 104 | -0.010 ( -0.027, 0.0074) | 0.26 | 149.03 (103) | 0.0021 | NA | NA | -0.039 ( -0.071, -0.0078) | 0.016 | -0.019 ( -0.042, 0.0041) | 0.11 | -0.021 ( -0.061, 0.020) | 0.32 | 0.0014 (0.0024) | 0.57 | 148.55 (102) | 0.0018 | -0.012 ( -0.030, 0.0059) | 0.19 | | |
|  | 3 | 100 | -0.014 ( -0.030, 0.0014) | 0.074 | 113.58 (99) | 0.15 | NA | NA | -0.042 ( -0.078, -0.0054) | 0.027 | -0.028 ( -0.051, -0.0044) | 0.020 | -0.022 ( -0.058, 0.015) | 0.24 | 0.00094 (0.0021) | 0.66 | 113.36 (98) | 0.14 | -0.015 ( -0.032, 0.0021) | 0.086 | | |
| IBD and LS-BMD(R*) | 1 | 104 | 0.0056 ( -0.015, 0.026) | 0.59 | 151.15 (103) | 0.0014 | NA | NA | -0.020 ( -0.056, 0.016) | 0.27 | -0.022 ( -0.052, 0.0085) | 0.16 | -0.017 ( -0.064, 0.030) | 0.47 | 0.0029 (0.0028) | 0.29 | 149.51 (102) | 0.0015 | 0.0052 ( -0.015, 0.026) | 0.62 | | |
|  | 3 | 101 | 0.00062 ( -0.018, 0.020) | 0.95 | 122.96 (100) | 0.059 | NA | NA | -0.021 ( -0.056, 0.015) | 0.27 | -0.022 ( -0.051, 0.0076) | 0.15 | -0.030 ( -0.073, 0.013) | 0.18 | 0.0039 (0.0025) | 0.13 | 120.13 (99) | 0.073 | 0.0023 ( -0.018, 0.022) | 0.82 | | |
| IBD and FA-BMD(R*) | 1 | 109 | -0.023 (-0.058,0.011) | 0.19 | 153.39 (108) | 0.0027 | -0.027 (0.017) | 0.12 | -0.011 (-0.080,0.058) | 0.75 | -0.015 (-0.061,0.032) | 0.53 | -0.036 (-0.12,0.047) | 0.39 | 0.0016 (0.0049) | 0.74 | 153.23 (107) | 0.0023 | -0.027 (-0.062,0.0087) | 0.14 | | |
|  | 2 | 107 | -0.020 (-0.053,0.012) | 0.22 | 131.07 (106) | 0.050 | NA | NA | -0.013 (-0.075,0.049) | 0.68 | -0.010 (-0.056,0.035) | 0.65 | -0.014 (-0.092,0.064) | 0.73 | -0.00084 (0.0046) | 0.85 | 131.03 (105) | 0.044 | -0.025 (-0.059,0.0098) | 0.16 | | |
|  | 3 | 105 | -0.026 (-0.058,0.0049) | 0.098 | 116.14 (104) | 0.20 | NA | NA | -0.012 (-0.082,0.058) | 0.75 | -0.011 (-0.059,0.037) | 0.65 | -0.0017 (-0.076,0.073) | 0.96 | -0.0031 (0.0044) | 0.48 | 115.56 (103) | 0.19 | -0.028 (-0.062,0.0051) | 0.097 | | |

Step^#^: 1, MR analysis with the complete selected SNPs; 2, MR analysis after removing the SNPs (with P value less than threshold in MR-PRESSO outlier test); 3, MR analysis after removing all the SNPs (with P value less than 1 in MR-PRESSO outlier test); 4, MR analysis after excluding the influential SNPs identified in ‘leave-one-out’ sensitivity analysis; R*, in replication practice; TB-BMD: Total Body-bone mineral density; FN-BMD: Femoral Neck bone mineral density; LS-BMD: Lumbar Spine bone mineral density; FA-BMD: Forearm bone mineral density; β, beta coefficient; Se, standard error; SNP, single nucleotide polymorphism; MR, Mendelian randomization; IVW: inverse variance weighting; MR-PRESSO: MR-Pleiotropy RESidual Sum and Outlier method; MBE: mode based estimate method; WMM: weighted median method; MR.RAPS: Robust Adjusted Profile Score; NA, not available.

Ibdfn leave one out analysis: 结果不稳定额

Ribdfn leave one out analysis 结果也是不稳定
