# Supplementary figures and images for: Mendelian randomization study of inflammatory bowel disease and bone mineral density
Source: BMC Med. 2020 Nov 10;18:312. doi: 10.1186/s12916-020-01778-5 (PMC7654011; doi:10.1186/s12916-020-01778-5)

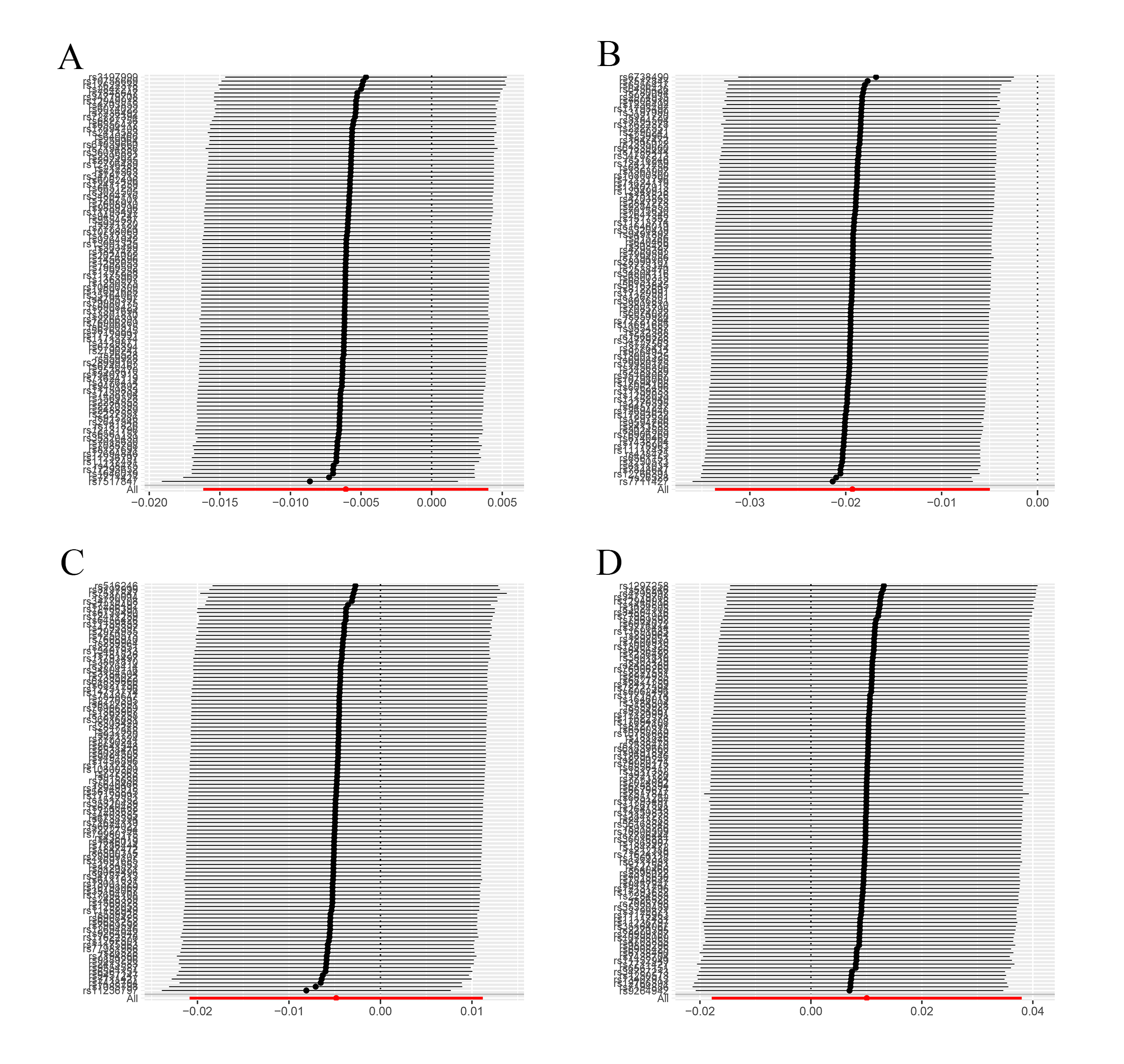

Supplement: Supplementary file 3 — Additional file 3: Figure S1. Funnel plots for MR analyses of the causal effect of IBD on BMDs in initial practice (A) TB-BMD (B) FN-BMD (C) LS-BMD (D) FA-BMD. Figure S2. Plots of “leave-one-out” analyses for MR analyses of the causal effect of IBD on BMDs in initial practice (A) TB-BMD (B) FN-BMD (C) LS-BMD (D) FA-BMD. Figure S3. Funnel plots for MR analyses of the causal effect of IBD on BMDs in replicative practice (A) TB-BMD (B) FN-BMD (C) LS-BMD (D) FA-BMD. Figure S4. Plots of “leave-one-out” analyses for MR analyses of the causal effect of IBD on BMDs in replicative practice (A) TB-BMD (B) FN-BMD (C) LS-BMD (D) FA-BMD. Figure S5. Funnel plots for MR analyses of the causal effect of UC on BMDs in initial practice (A) TB-BMD (B) FN-BMD (C) LS-BMD (D) FA-BMD. Figure S6. Plots of “leave-one-out” analyses for MR analyses of the causal effect of UC on BMDs in initial practice (A) TB-BMD (B) FN-BMD (C) LS-BMD (D) FA-BMD. Figure S7. Funnel plots for MR analyses of the causal effect of UC on BMDs in replicative practice (A) TB-BMD (B) FN-BMD (C) LS-BMD (D) FA-BMD. Figure S8. Plots of “leave-one-out” analyses for MR analyses of the causal effect of UC on BMDs in replicative practice (A) TB-BMD (B) FN-BMD (C) LS-BMD (D) FA-BMD. Figure S9. Funnel plots for MR analyses of the causal effect of CD on BMDs in initial practice (A) TB-BMD (B) FN-BMD (C) LS-BMD (D) FA-BMD. Figure S10. Plots of “leave-one-out” analyses for MR analyses of the causal effect of CD on BMDs in initial practice (A) TB-BMD (B) FN-BMD (C) LS-BMD (D) FA-BMD. Figure S11. Funnel plots for MR analyses of the causal effect of CD on BMDs in replicative practice (A) TB-BMD (B) FN-BMD (C) LS-BMD (D) FA-BMD. Figure S12. Plots of “leave-one-out” analyses for MR analyses of the causal effect of CD on BMDs in replicative practice (A) TB-BMD (B) FN-BMD (C) LS-BMD (D) FA-BMD. [file 12916_2020_1778_MOESM3_ESM.zip › Additional File3 Figure S10R2.tif]

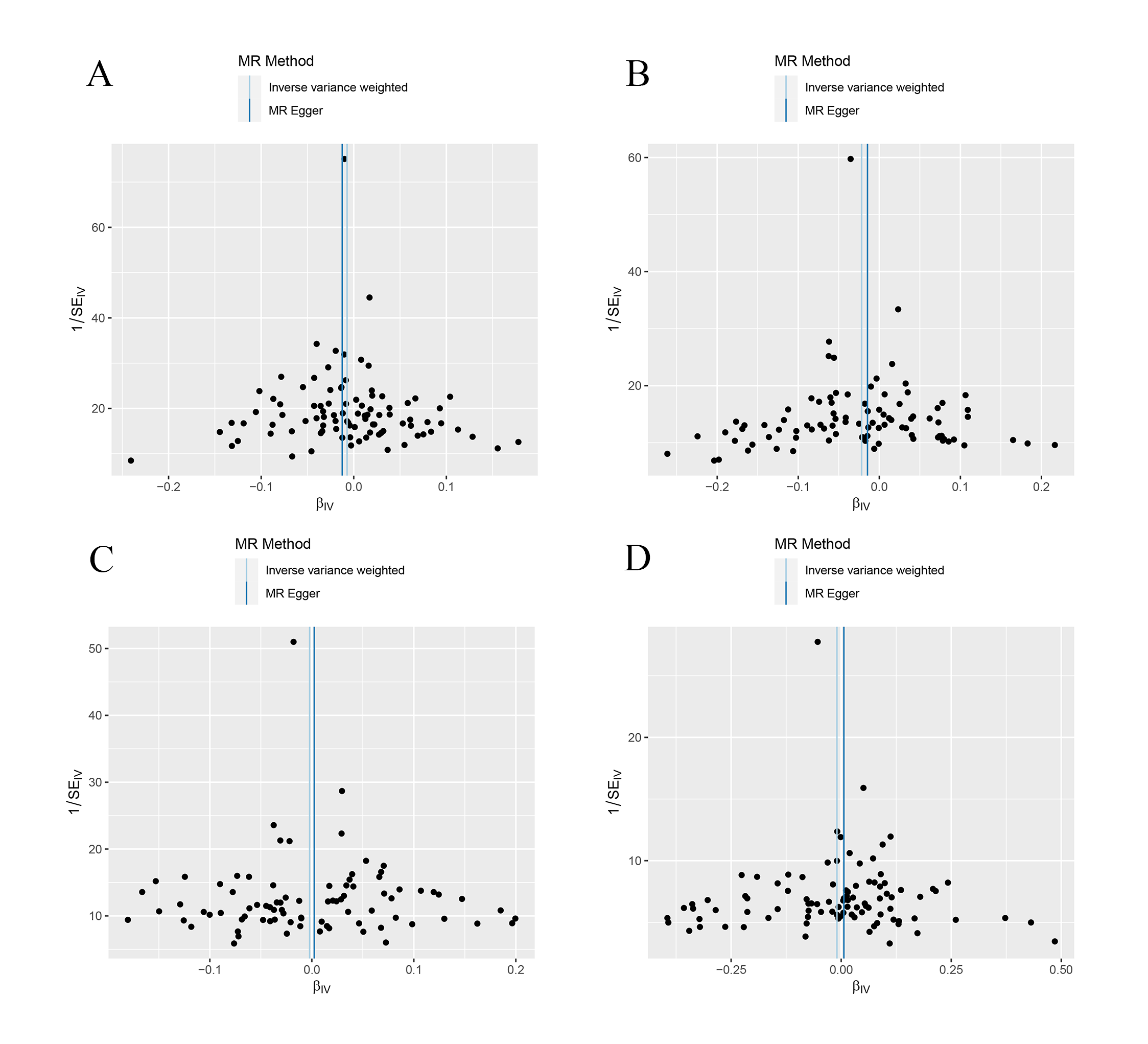

Supplement: Supplementary file 3 — Additional file 3: Figure S1. Funnel plots for MR analyses of the causal effect of IBD on BMDs in initial practice (A) TB-BMD (B) FN-BMD (C) LS-BMD (D) FA-BMD. Figure S2. Plots of “leave-one-out” analyses for MR analyses of the causal effect of IBD on BMDs in initial practice (A) TB-BMD (B) FN-BMD (C) LS-BMD (D) FA-BMD. Figure S3. Funnel plots for MR analyses of the causal effect of IBD on BMDs in replicative practice (A) TB-BMD (B) FN-BMD (C) LS-BMD (D) FA-BMD. Figure S4. Plots of “leave-one-out” analyses for MR analyses of the causal effect of IBD on BMDs in replicative practice (A) TB-BMD (B) FN-BMD (C) LS-BMD (D) FA-BMD. Figure S5. Funnel plots for MR analyses of the causal effect of UC on BMDs in initial practice (A) TB-BMD (B) FN-BMD (C) LS-BMD (D) FA-BMD. Figure S6. Plots of “leave-one-out” analyses for MR analyses of the causal effect of UC on BMDs in initial practice (A) TB-BMD (B) FN-BMD (C) LS-BMD (D) FA-BMD. Figure S7. Funnel plots for MR analyses of the causal effect of UC on BMDs in replicative practice (A) TB-BMD (B) FN-BMD (C) LS-BMD (D) FA-BMD. Figure S8. Plots of “leave-one-out” analyses for MR analyses of the causal effect of UC on BMDs in replicative practice (A) TB-BMD (B) FN-BMD (C) LS-BMD (D) FA-BMD. Figure S9. Funnel plots for MR analyses of the causal effect of CD on BMDs in initial practice (A) TB-BMD (B) FN-BMD (C) LS-BMD (D) FA-BMD. Figure S10. Plots of “leave-one-out” analyses for MR analyses of the causal effect of CD on BMDs in initial practice (A) TB-BMD (B) FN-BMD (C) LS-BMD (D) FA-BMD. Figure S11. Funnel plots for MR analyses of the causal effect of CD on BMDs in replicative practice (A) TB-BMD (B) FN-BMD (C) LS-BMD (D) FA-BMD. Figure S12. Plots of “leave-one-out” analyses for MR analyses of the causal effect of CD on BMDs in replicative practice (A) TB-BMD (B) FN-BMD (C) LS-BMD (D) FA-BMD. [file 12916_2020_1778_MOESM3_ESM.zip › Additional File3 Figure S11R2.tif]

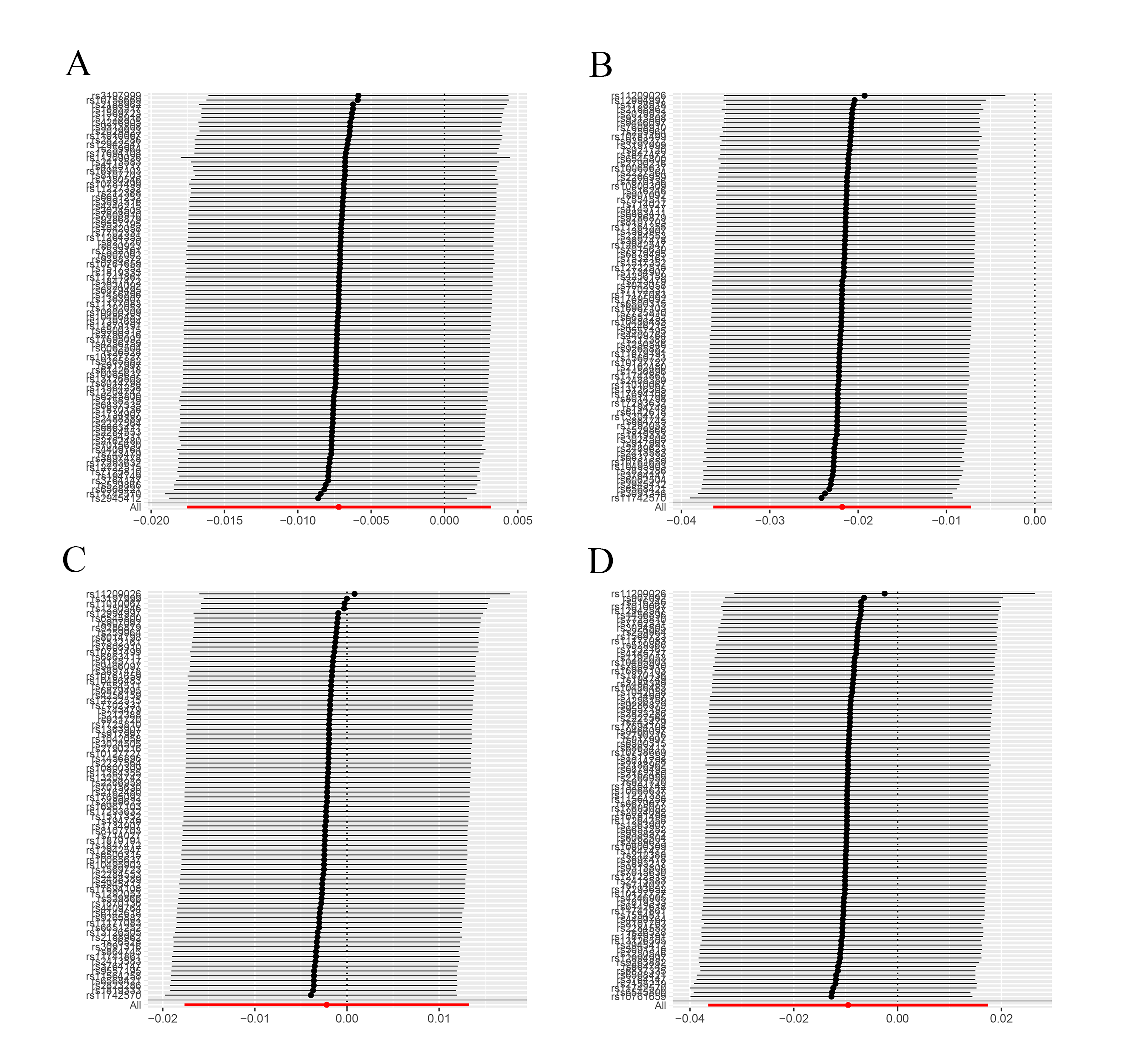

Supplement: Supplementary file 3 — Additional file 3: Figure S1. Funnel plots for MR analyses of the causal effect of IBD on BMDs in initial practice (A) TB-BMD (B) FN-BMD (C) LS-BMD (D) FA-BMD. Figure S2. Plots of “leave-one-out” analyses for MR analyses of the causal effect of IBD on BMDs in initial practice (A) TB-BMD (B) FN-BMD (C) LS-BMD (D) FA-BMD. Figure S3. Funnel plots for MR analyses of the causal effect of IBD on BMDs in replicative practice (A) TB-BMD (B) FN-BMD (C) LS-BMD (D) FA-BMD. Figure S4. Plots of “leave-one-out” analyses for MR analyses of the causal effect of IBD on BMDs in replicative practice (A) TB-BMD (B) FN-BMD (C) LS-BMD (D) FA-BMD. Figure S5. Funnel plots for MR analyses of the causal effect of UC on BMDs in initial practice (A) TB-BMD (B) FN-BMD (C) LS-BMD (D) FA-BMD. Figure S6. Plots of “leave-one-out” analyses for MR analyses of the causal effect of UC on BMDs in initial practice (A) TB-BMD (B) FN-BMD (C) LS-BMD (D) FA-BMD. Figure S7. Funnel plots for MR analyses of the causal effect of UC on BMDs in replicative practice (A) TB-BMD (B) FN-BMD (C) LS-BMD (D) FA-BMD. Figure S8. Plots of “leave-one-out” analyses for MR analyses of the causal effect of UC on BMDs in replicative practice (A) TB-BMD (B) FN-BMD (C) LS-BMD (D) FA-BMD. Figure S9. Funnel plots for MR analyses of the causal effect of CD on BMDs in initial practice (A) TB-BMD (B) FN-BMD (C) LS-BMD (D) FA-BMD. Figure S10. Plots of “leave-one-out” analyses for MR analyses of the causal effect of CD on BMDs in initial practice (A) TB-BMD (B) FN-BMD (C) LS-BMD (D) FA-BMD. Figure S11. Funnel plots for MR analyses of the causal effect of CD on BMDs in replicative practice (A) TB-BMD (B) FN-BMD (C) LS-BMD (D) FA-BMD. Figure S12. Plots of “leave-one-out” analyses for MR analyses of the causal effect of CD on BMDs in replicative practice (A) TB-BMD (B) FN-BMD (C) LS-BMD (D) FA-BMD. [file 12916_2020_1778_MOESM3_ESM.zip › Additional File3 Figure S12R2.tif]

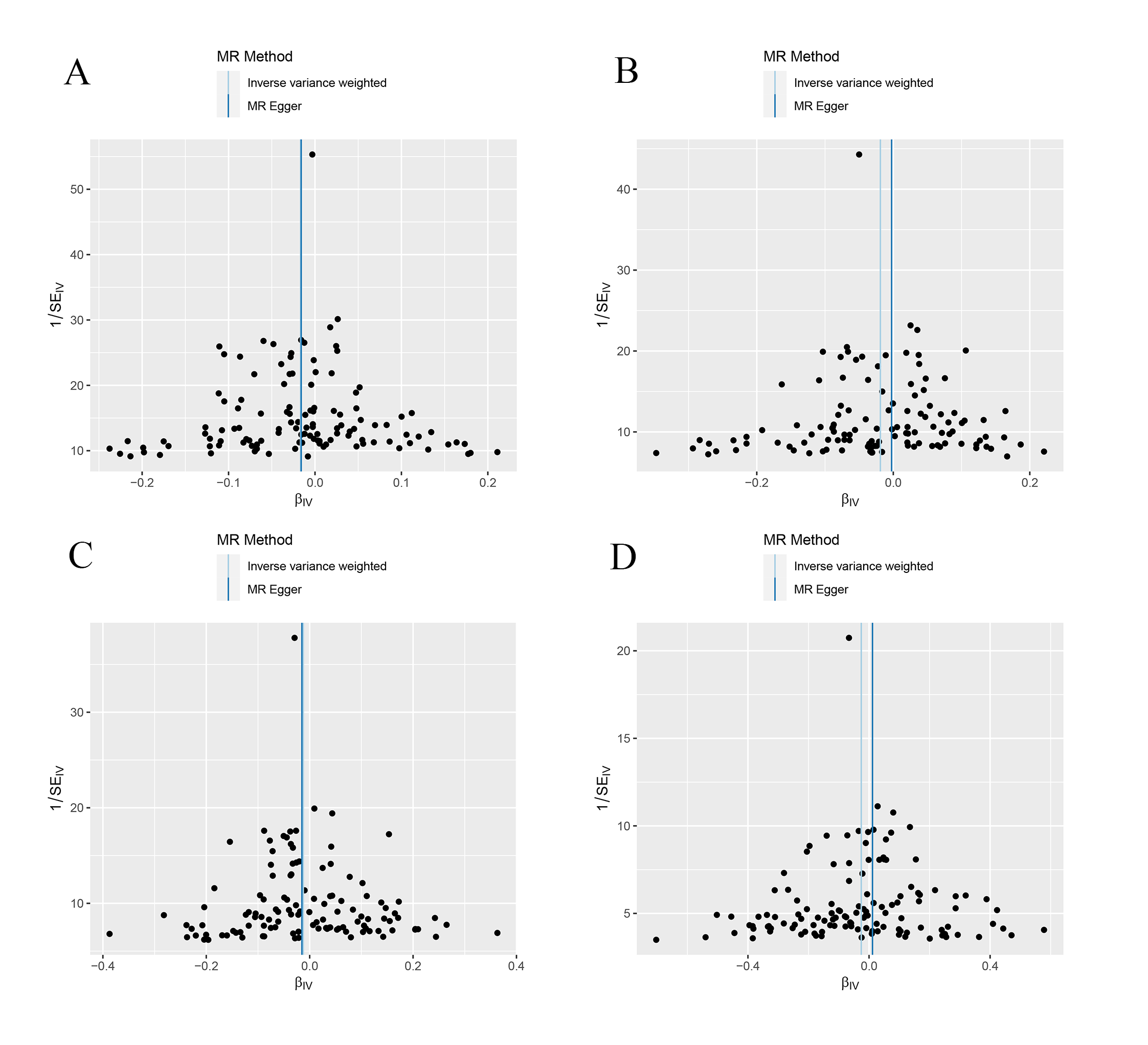

Supplement: Supplementary file 3 — Additional file 3: Figure S1. Funnel plots for MR analyses of the causal effect of IBD on BMDs in initial practice (A) TB-BMD (B) FN-BMD (C) LS-BMD (D) FA-BMD. Figure S2. Plots of “leave-one-out” analyses for MR analyses of the causal effect of IBD on BMDs in initial practice (A) TB-BMD (B) FN-BMD (C) LS-BMD (D) FA-BMD. Figure S3. Funnel plots for MR analyses of the causal effect of IBD on BMDs in replicative practice (A) TB-BMD (B) FN-BMD (C) LS-BMD (D) FA-BMD. Figure S4. Plots of “leave-one-out” analyses for MR analyses of the causal effect of IBD on BMDs in replicative practice (A) TB-BMD (B) FN-BMD (C) LS-BMD (D) FA-BMD. Figure S5. Funnel plots for MR analyses of the causal effect of UC on BMDs in initial practice (A) TB-BMD (B) FN-BMD (C) LS-BMD (D) FA-BMD. Figure S6. Plots of “leave-one-out” analyses for MR analyses of the causal effect of UC on BMDs in initial practice (A) TB-BMD (B) FN-BMD (C) LS-BMD (D) FA-BMD. Figure S7. Funnel plots for MR analyses of the causal effect of UC on BMDs in replicative practice (A) TB-BMD (B) FN-BMD (C) LS-BMD (D) FA-BMD. Figure S8. Plots of “leave-one-out” analyses for MR analyses of the causal effect of UC on BMDs in replicative practice (A) TB-BMD (B) FN-BMD (C) LS-BMD (D) FA-BMD. Figure S9. Funnel plots for MR analyses of the causal effect of CD on BMDs in initial practice (A) TB-BMD (B) FN-BMD (C) LS-BMD (D) FA-BMD. Figure S10. Plots of “leave-one-out” analyses for MR analyses of the causal effect of CD on BMDs in initial practice (A) TB-BMD (B) FN-BMD (C) LS-BMD (D) FA-BMD. Figure S11. Funnel plots for MR analyses of the causal effect of CD on BMDs in replicative practice (A) TB-BMD (B) FN-BMD (C) LS-BMD (D) FA-BMD. Figure S12. Plots of “leave-one-out” analyses for MR analyses of the causal effect of CD on BMDs in replicative practice (A) TB-BMD (B) FN-BMD (C) LS-BMD (D) FA-BMD. [file 12916_2020_1778_MOESM3_ESM.zip › Additional File3 Figure S1R2.tif]

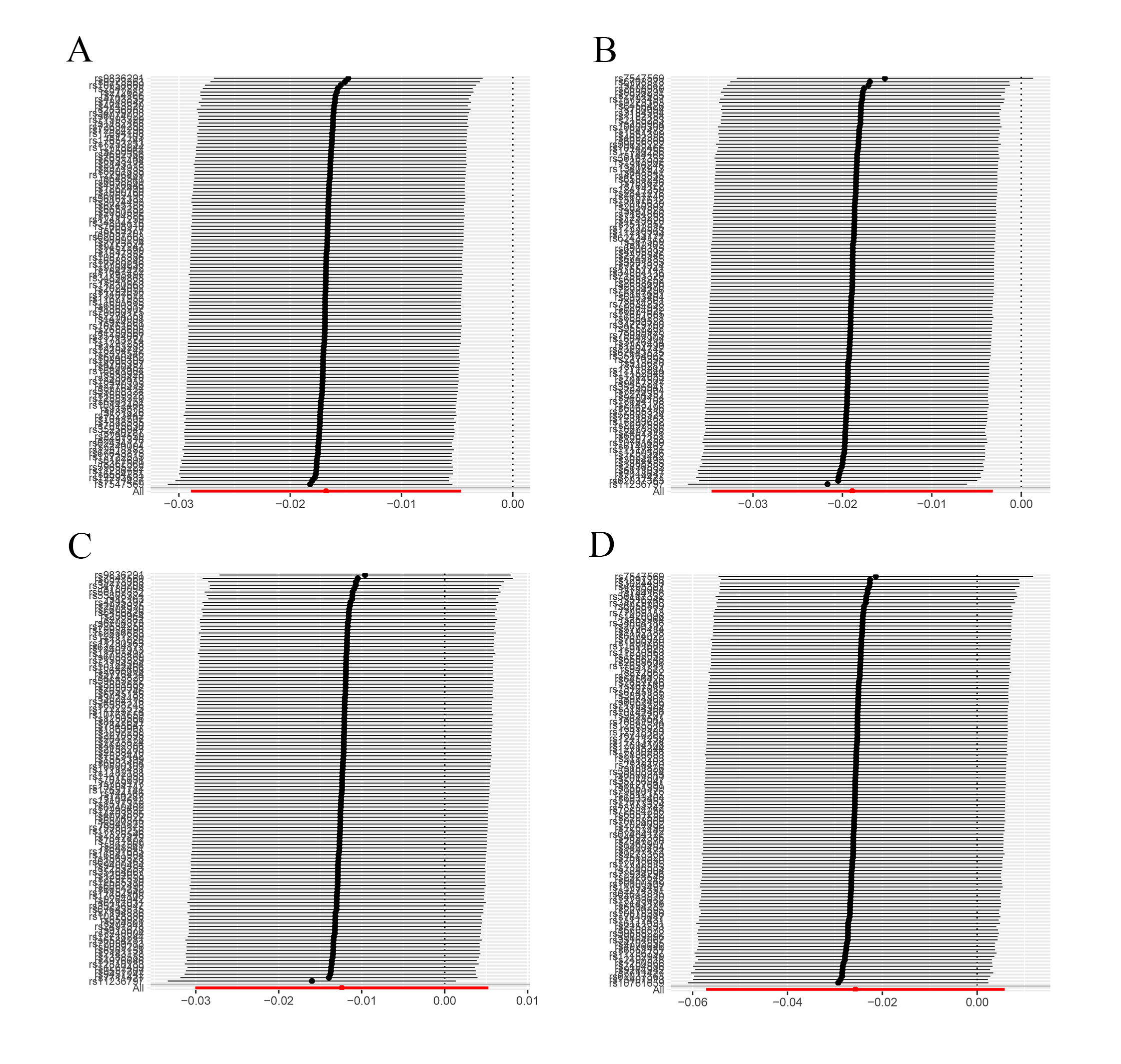

Supplement: Supplementary file 3 — Additional file 3: Figure S1. Funnel plots for MR analyses of the causal effect of IBD on BMDs in initial practice (A) TB-BMD (B) FN-BMD (C) LS-BMD (D) FA-BMD. Figure S2. Plots of “leave-one-out” analyses for MR analyses of the causal effect of IBD on BMDs in initial practice (A) TB-BMD (B) FN-BMD (C) LS-BMD (D) FA-BMD. Figure S3. Funnel plots for MR analyses of the causal effect of IBD on BMDs in replicative practice (A) TB-BMD (B) FN-BMD (C) LS-BMD (D) FA-BMD. Figure S4. Plots of “leave-one-out” analyses for MR analyses of the causal effect of IBD on BMDs in replicative practice (A) TB-BMD (B) FN-BMD (C) LS-BMD (D) FA-BMD. Figure S5. Funnel plots for MR analyses of the causal effect of UC on BMDs in initial practice (A) TB-BMD (B) FN-BMD (C) LS-BMD (D) FA-BMD. Figure S6. Plots of “leave-one-out” analyses for MR analyses of the causal effect of UC on BMDs in initial practice (A) TB-BMD (B) FN-BMD (C) LS-BMD (D) FA-BMD. Figure S7. Funnel plots for MR analyses of the causal effect of UC on BMDs in replicative practice (A) TB-BMD (B) FN-BMD (C) LS-BMD (D) FA-BMD. Figure S8. Plots of “leave-one-out” analyses for MR analyses of the causal effect of UC on BMDs in replicative practice (A) TB-BMD (B) FN-BMD (C) LS-BMD (D) FA-BMD. Figure S9. Funnel plots for MR analyses of the causal effect of CD on BMDs in initial practice (A) TB-BMD (B) FN-BMD (C) LS-BMD (D) FA-BMD. Figure S10. Plots of “leave-one-out” analyses for MR analyses of the causal effect of CD on BMDs in initial practice (A) TB-BMD (B) FN-BMD (C) LS-BMD (D) FA-BMD. Figure S11. Funnel plots for MR analyses of the causal effect of CD on BMDs in replicative practice (A) TB-BMD (B) FN-BMD (C) LS-BMD (D) FA-BMD. Figure S12. Plots of “leave-one-out” analyses for MR analyses of the causal effect of CD on BMDs in replicative practice (A) TB-BMD (B) FN-BMD (C) LS-BMD (D) FA-BMD. [file 12916_2020_1778_MOESM3_ESM.zip › Additional File3 Figure S2R2.tif]

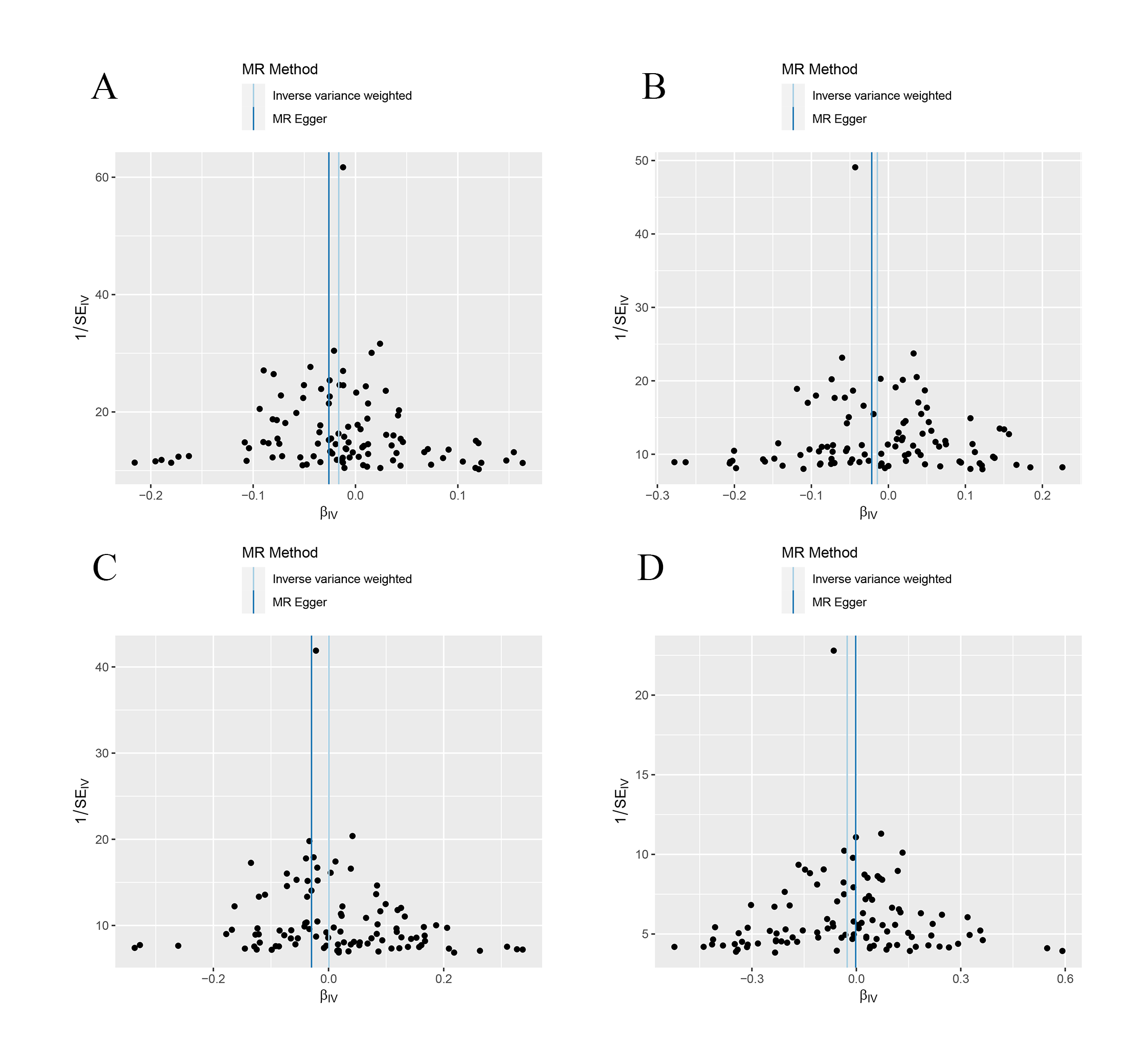

Supplement: Supplementary file 3 — Additional file 3: Figure S1. Funnel plots for MR analyses of the causal effect of IBD on BMDs in initial practice (A) TB-BMD (B) FN-BMD (C) LS-BMD (D) FA-BMD. Figure S2. Plots of “leave-one-out” analyses for MR analyses of the causal effect of IBD on BMDs in initial practice (A) TB-BMD (B) FN-BMD (C) LS-BMD (D) FA-BMD. Figure S3. Funnel plots for MR analyses of the causal effect of IBD on BMDs in replicative practice (A) TB-BMD (B) FN-BMD (C) LS-BMD (D) FA-BMD. Figure S4. Plots of “leave-one-out” analyses for MR analyses of the causal effect of IBD on BMDs in replicative practice (A) TB-BMD (B) FN-BMD (C) LS-BMD (D) FA-BMD. Figure S5. Funnel plots for MR analyses of the causal effect of UC on BMDs in initial practice (A) TB-BMD (B) FN-BMD (C) LS-BMD (D) FA-BMD. Figure S6. Plots of “leave-one-out” analyses for MR analyses of the causal effect of UC on BMDs in initial practice (A) TB-BMD (B) FN-BMD (C) LS-BMD (D) FA-BMD. Figure S7. Funnel plots for MR analyses of the causal effect of UC on BMDs in replicative practice (A) TB-BMD (B) FN-BMD (C) LS-BMD (D) FA-BMD. Figure S8. Plots of “leave-one-out” analyses for MR analyses of the causal effect of UC on BMDs in replicative practice (A) TB-BMD (B) FN-BMD (C) LS-BMD (D) FA-BMD. Figure S9. Funnel plots for MR analyses of the causal effect of CD on BMDs in initial practice (A) TB-BMD (B) FN-BMD (C) LS-BMD (D) FA-BMD. Figure S10. Plots of “leave-one-out” analyses for MR analyses of the causal effect of CD on BMDs in initial practice (A) TB-BMD (B) FN-BMD (C) LS-BMD (D) FA-BMD. Figure S11. Funnel plots for MR analyses of the causal effect of CD on BMDs in replicative practice (A) TB-BMD (B) FN-BMD (C) LS-BMD (D) FA-BMD. Figure S12. Plots of “leave-one-out” analyses for MR analyses of the causal effect of CD on BMDs in replicative practice (A) TB-BMD (B) FN-BMD (C) LS-BMD (D) FA-BMD. [file 12916_2020_1778_MOESM3_ESM.zip › Additional File3 Figure S3R2.tif]

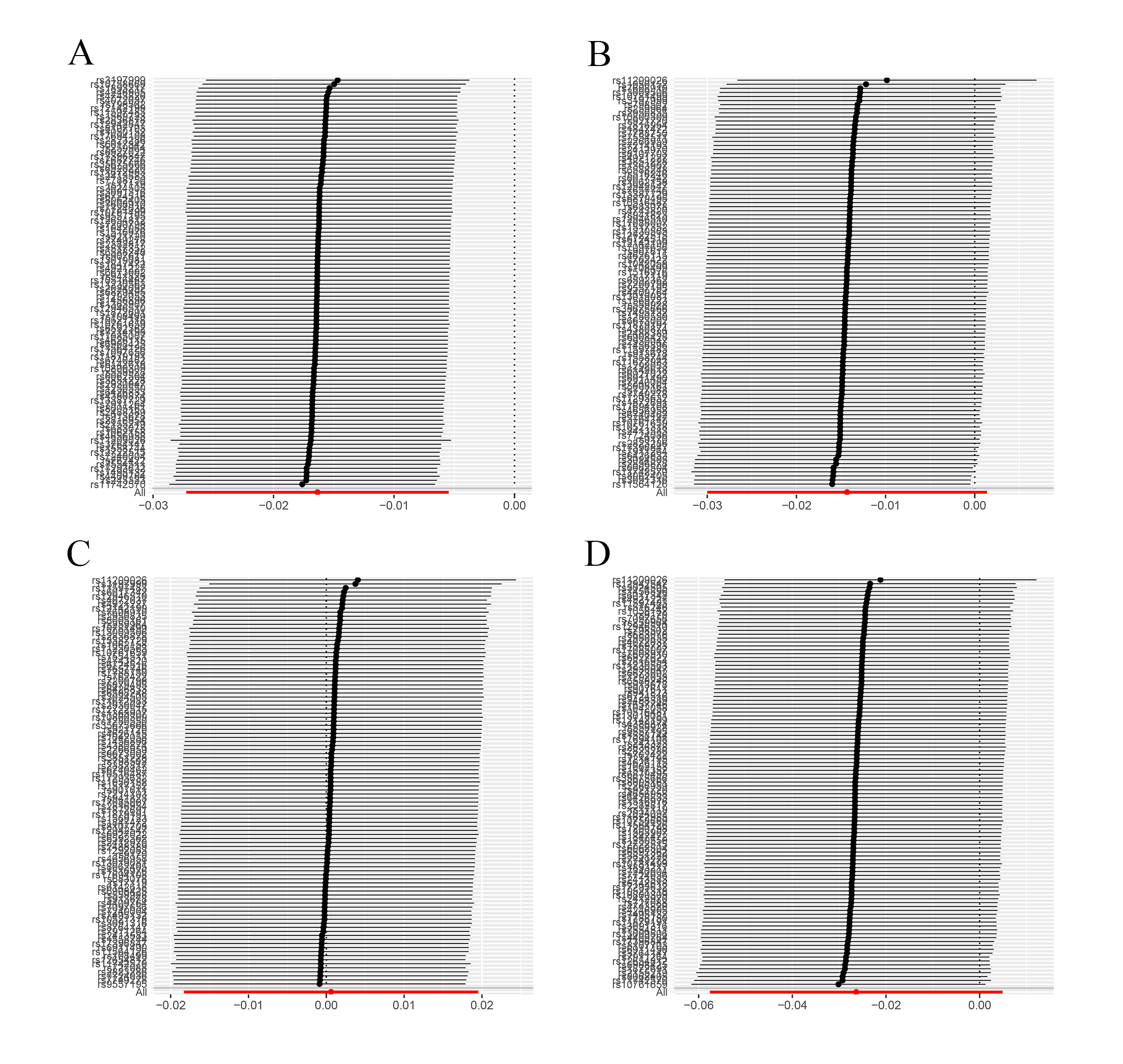

Supplement: Supplementary file 3 — Additional file 3: Figure S1. Funnel plots for MR analyses of the causal effect of IBD on BMDs in initial practice (A) TB-BMD (B) FN-BMD (C) LS-BMD (D) FA-BMD. Figure S2. Plots of “leave-one-out” analyses for MR analyses of the causal effect of IBD on BMDs in initial practice (A) TB-BMD (B) FN-BMD (C) LS-BMD (D) FA-BMD. Figure S3. Funnel plots for MR analyses of the causal effect of IBD on BMDs in replicative practice (A) TB-BMD (B) FN-BMD (C) LS-BMD (D) FA-BMD. Figure S4. Plots of “leave-one-out” analyses for MR analyses of the causal effect of IBD on BMDs in replicative practice (A) TB-BMD (B) FN-BMD (C) LS-BMD (D) FA-BMD. Figure S5. Funnel plots for MR analyses of the causal effect of UC on BMDs in initial practice (A) TB-BMD (B) FN-BMD (C) LS-BMD (D) FA-BMD. Figure S6. Plots of “leave-one-out” analyses for MR analyses of the causal effect of UC on BMDs in initial practice (A) TB-BMD (B) FN-BMD (C) LS-BMD (D) FA-BMD. Figure S7. Funnel plots for MR analyses of the causal effect of UC on BMDs in replicative practice (A) TB-BMD (B) FN-BMD (C) LS-BMD (D) FA-BMD. Figure S8. Plots of “leave-one-out” analyses for MR analyses of the causal effect of UC on BMDs in replicative practice (A) TB-BMD (B) FN-BMD (C) LS-BMD (D) FA-BMD. Figure S9. Funnel plots for MR analyses of the causal effect of CD on BMDs in initial practice (A) TB-BMD (B) FN-BMD (C) LS-BMD (D) FA-BMD. Figure S10. Plots of “leave-one-out” analyses for MR analyses of the causal effect of CD on BMDs in initial practice (A) TB-BMD (B) FN-BMD (C) LS-BMD (D) FA-BMD. Figure S11. Funnel plots for MR analyses of the causal effect of CD on BMDs in replicative practice (A) TB-BMD (B) FN-BMD (C) LS-BMD (D) FA-BMD. Figure S12. Plots of “leave-one-out” analyses for MR analyses of the causal effect of CD on BMDs in replicative practice (A) TB-BMD (B) FN-BMD (C) LS-BMD (D) FA-BMD. [file 12916_2020_1778_MOESM3_ESM.zip › Additional File3 Figure S4R2.tif]

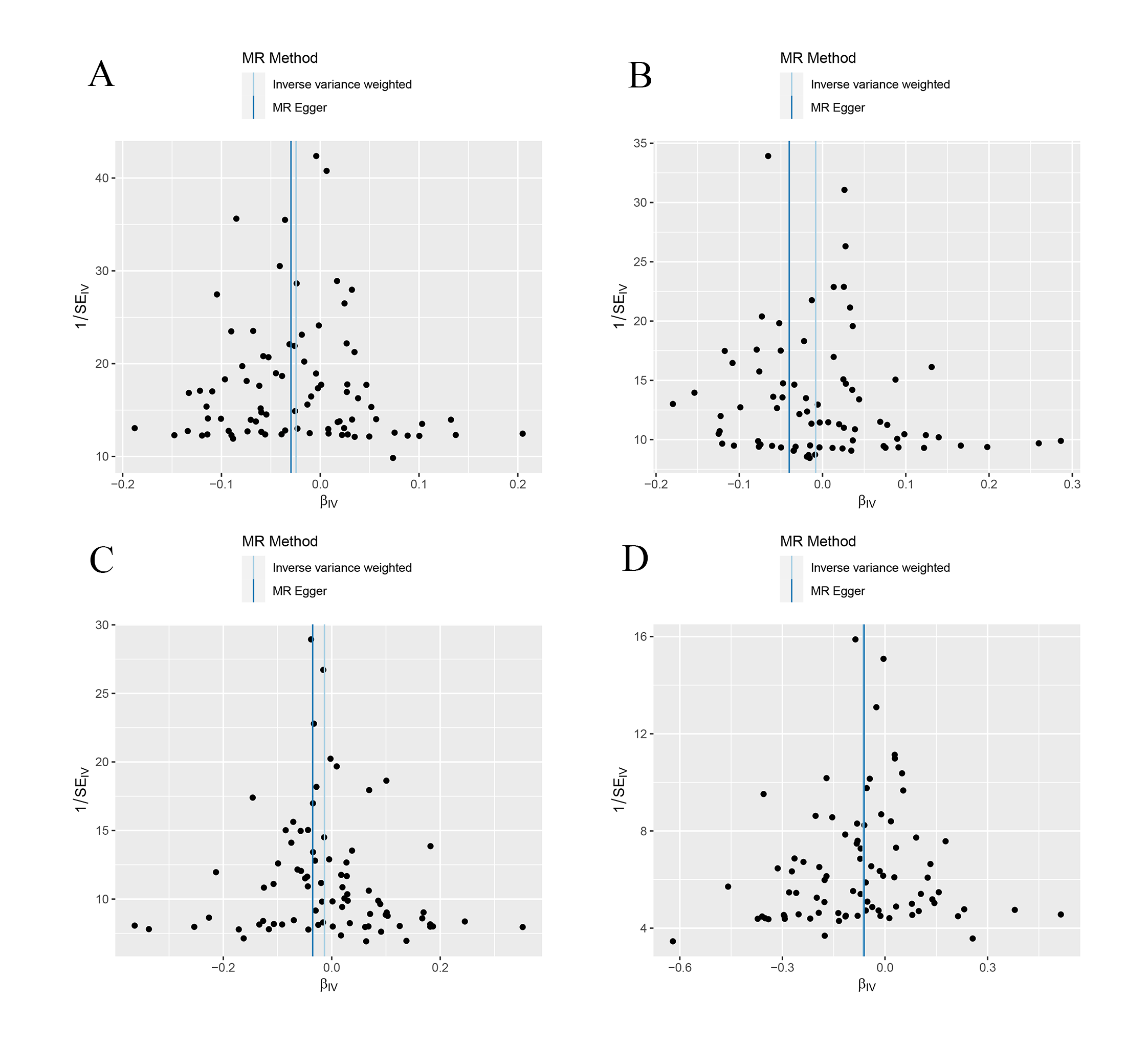

Supplement: Supplementary file 3 — Additional file 3: Figure S1. Funnel plots for MR analyses of the causal effect of IBD on BMDs in initial practice (A) TB-BMD (B) FN-BMD (C) LS-BMD (D) FA-BMD. Figure S2. Plots of “leave-one-out” analyses for MR analyses of the causal effect of IBD on BMDs in initial practice (A) TB-BMD (B) FN-BMD (C) LS-BMD (D) FA-BMD. Figure S3. Funnel plots for MR analyses of the causal effect of IBD on BMDs in replicative practice (A) TB-BMD (B) FN-BMD (C) LS-BMD (D) FA-BMD. Figure S4. Plots of “leave-one-out” analyses for MR analyses of the causal effect of IBD on BMDs in replicative practice (A) TB-BMD (B) FN-BMD (C) LS-BMD (D) FA-BMD. Figure S5. Funnel plots for MR analyses of the causal effect of UC on BMDs in initial practice (A) TB-BMD (B) FN-BMD (C) LS-BMD (D) FA-BMD. Figure S6. Plots of “leave-one-out” analyses for MR analyses of the causal effect of UC on BMDs in initial practice (A) TB-BMD (B) FN-BMD (C) LS-BMD (D) FA-BMD. Figure S7. Funnel plots for MR analyses of the causal effect of UC on BMDs in replicative practice (A) TB-BMD (B) FN-BMD (C) LS-BMD (D) FA-BMD. Figure S8. Plots of “leave-one-out” analyses for MR analyses of the causal effect of UC on BMDs in replicative practice (A) TB-BMD (B) FN-BMD (C) LS-BMD (D) FA-BMD. Figure S9. Funnel plots for MR analyses of the causal effect of CD on BMDs in initial practice (A) TB-BMD (B) FN-BMD (C) LS-BMD (D) FA-BMD. Figure S10. Plots of “leave-one-out” analyses for MR analyses of the causal effect of CD on BMDs in initial practice (A) TB-BMD (B) FN-BMD (C) LS-BMD (D) FA-BMD. Figure S11. Funnel plots for MR analyses of the causal effect of CD on BMDs in replicative practice (A) TB-BMD (B) FN-BMD (C) LS-BMD (D) FA-BMD. Figure S12. Plots of “leave-one-out” analyses for MR analyses of the causal effect of CD on BMDs in replicative practice (A) TB-BMD (B) FN-BMD (C) LS-BMD (D) FA-BMD. [file 12916_2020_1778_MOESM3_ESM.zip › Additional File3 Figure S5R2.tif]

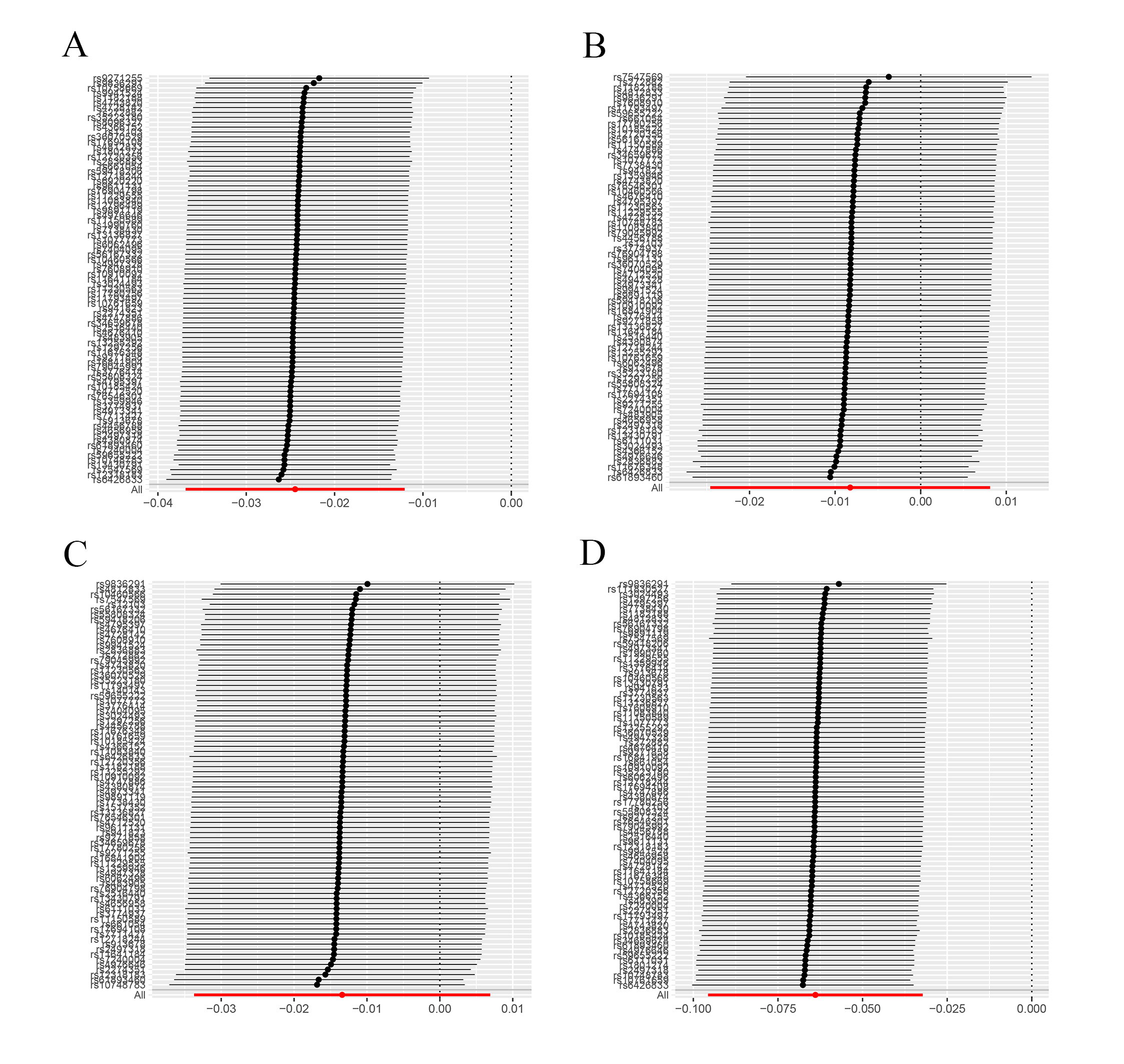

Supplement: Supplementary file 3 — Additional file 3: Figure S1. Funnel plots for MR analyses of the causal effect of IBD on BMDs in initial practice (A) TB-BMD (B) FN-BMD (C) LS-BMD (D) FA-BMD. Figure S2. Plots of “leave-one-out” analyses for MR analyses of the causal effect of IBD on BMDs in initial practice (A) TB-BMD (B) FN-BMD (C) LS-BMD (D) FA-BMD. Figure S3. Funnel plots for MR analyses of the causal effect of IBD on BMDs in replicative practice (A) TB-BMD (B) FN-BMD (C) LS-BMD (D) FA-BMD. Figure S4. Plots of “leave-one-out” analyses for MR analyses of the causal effect of IBD on BMDs in replicative practice (A) TB-BMD (B) FN-BMD (C) LS-BMD (D) FA-BMD. Figure S5. Funnel plots for MR analyses of the causal effect of UC on BMDs in initial practice (A) TB-BMD (B) FN-BMD (C) LS-BMD (D) FA-BMD. Figure S6. Plots of “leave-one-out” analyses for MR analyses of the causal effect of UC on BMDs in initial practice (A) TB-BMD (B) FN-BMD (C) LS-BMD (D) FA-BMD. Figure S7. Funnel plots for MR analyses of the causal effect of UC on BMDs in replicative practice (A) TB-BMD (B) FN-BMD (C) LS-BMD (D) FA-BMD. Figure S8. Plots of “leave-one-out” analyses for MR analyses of the causal effect of UC on BMDs in replicative practice (A) TB-BMD (B) FN-BMD (C) LS-BMD (D) FA-BMD. Figure S9. Funnel plots for MR analyses of the causal effect of CD on BMDs in initial practice (A) TB-BMD (B) FN-BMD (C) LS-BMD (D) FA-BMD. Figure S10. Plots of “leave-one-out” analyses for MR analyses of the causal effect of CD on BMDs in initial practice (A) TB-BMD (B) FN-BMD (C) LS-BMD (D) FA-BMD. Figure S11. Funnel plots for MR analyses of the causal effect of CD on BMDs in replicative practice (A) TB-BMD (B) FN-BMD (C) LS-BMD (D) FA-BMD. Figure S12. Plots of “leave-one-out” analyses for MR analyses of the causal effect of CD on BMDs in replicative practice (A) TB-BMD (B) FN-BMD (C) LS-BMD (D) FA-BMD. [file 12916_2020_1778_MOESM3_ESM.zip › Additional File3 Figure S6R2.tif]

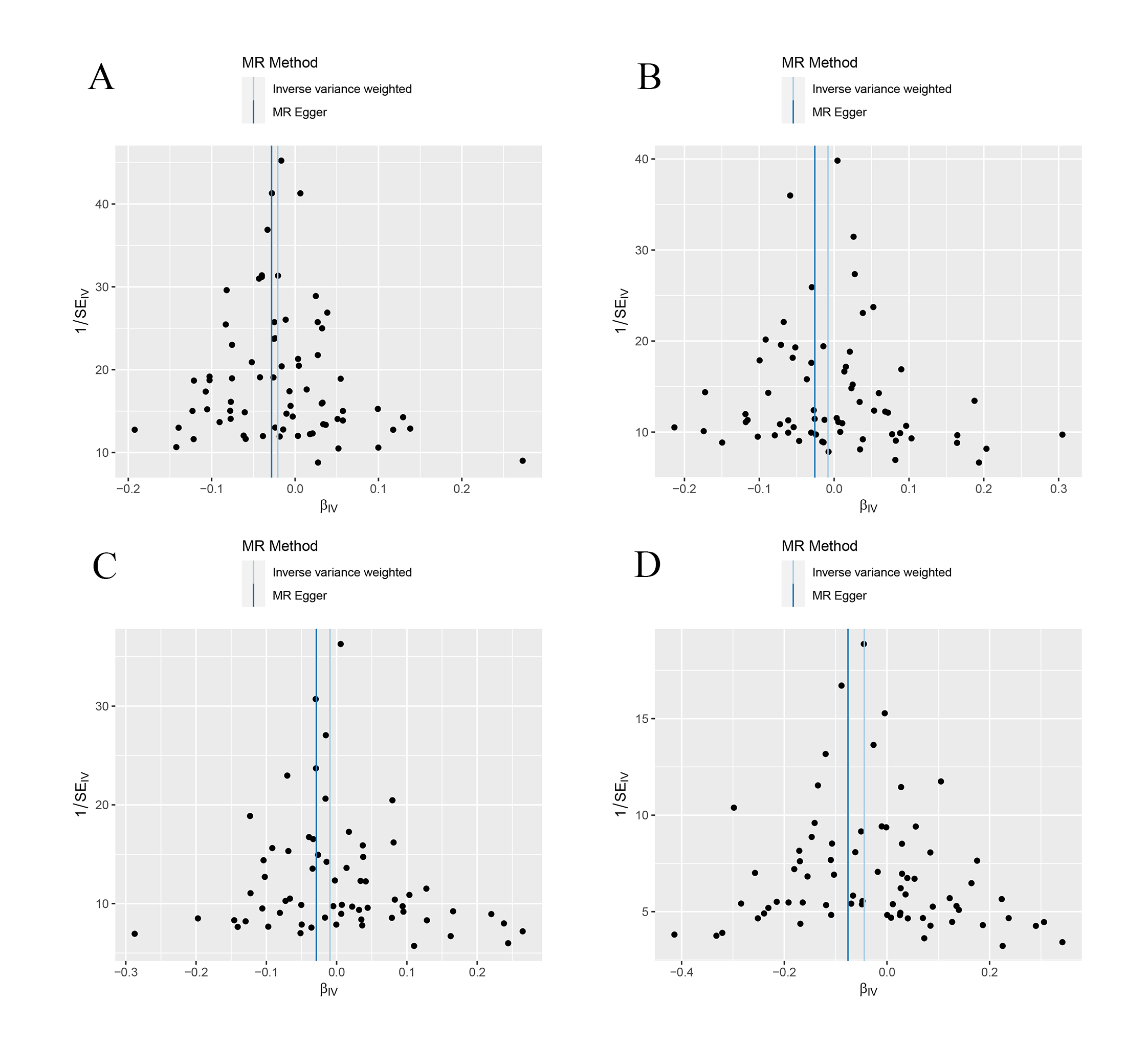

Supplement: Supplementary file 3 — Additional file 3: Figure S1. Funnel plots for MR analyses of the causal effect of IBD on BMDs in initial practice (A) TB-BMD (B) FN-BMD (C) LS-BMD (D) FA-BMD. Figure S2. Plots of “leave-one-out” analyses for MR analyses of the causal effect of IBD on BMDs in initial practice (A) TB-BMD (B) FN-BMD (C) LS-BMD (D) FA-BMD. Figure S3. Funnel plots for MR analyses of the causal effect of IBD on BMDs in replicative practice (A) TB-BMD (B) FN-BMD (C) LS-BMD (D) FA-BMD. Figure S4. Plots of “leave-one-out” analyses for MR analyses of the causal effect of IBD on BMDs in replicative practice (A) TB-BMD (B) FN-BMD (C) LS-BMD (D) FA-BMD. Figure S5. Funnel plots for MR analyses of the causal effect of UC on BMDs in initial practice (A) TB-BMD (B) FN-BMD (C) LS-BMD (D) FA-BMD. Figure S6. Plots of “leave-one-out” analyses for MR analyses of the causal effect of UC on BMDs in initial practice (A) TB-BMD (B) FN-BMD (C) LS-BMD (D) FA-BMD. Figure S7. Funnel plots for MR analyses of the causal effect of UC on BMDs in replicative practice (A) TB-BMD (B) FN-BMD (C) LS-BMD (D) FA-BMD. Figure S8. Plots of “leave-one-out” analyses for MR analyses of the causal effect of UC on BMDs in replicative practice (A) TB-BMD (B) FN-BMD (C) LS-BMD (D) FA-BMD. Figure S9. Funnel plots for MR analyses of the causal effect of CD on BMDs in initial practice (A) TB-BMD (B) FN-BMD (C) LS-BMD (D) FA-BMD. Figure S10. Plots of “leave-one-out” analyses for MR analyses of the causal effect of CD on BMDs in initial practice (A) TB-BMD (B) FN-BMD (C) LS-BMD (D) FA-BMD. Figure S11. Funnel plots for MR analyses of the causal effect of CD on BMDs in replicative practice (A) TB-BMD (B) FN-BMD (C) LS-BMD (D) FA-BMD. Figure S12. Plots of “leave-one-out” analyses for MR analyses of the causal effect of CD on BMDs in replicative practice (A) TB-BMD (B) FN-BMD (C) LS-BMD (D) FA-BMD. [file 12916_2020_1778_MOESM3_ESM.zip › Additional File3 Figure S7R2.tif]

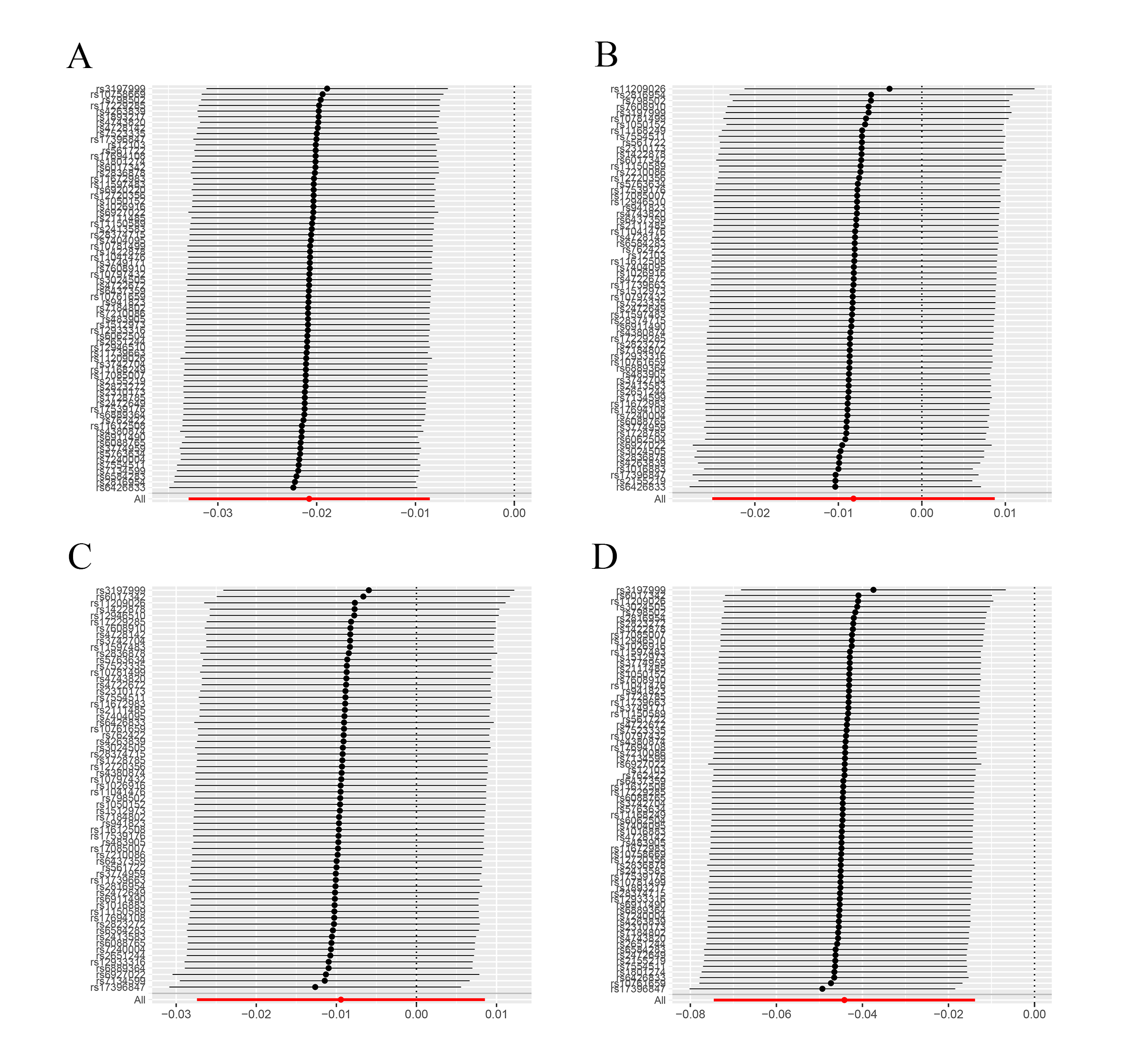

Supplement: Supplementary file 3 — Additional file 3: Figure S1. Funnel plots for MR analyses of the causal effect of IBD on BMDs in initial practice (A) TB-BMD (B) FN-BMD (C) LS-BMD (D) FA-BMD. Figure S2. Plots of “leave-one-out” analyses for MR analyses of the causal effect of IBD on BMDs in initial practice (A) TB-BMD (B) FN-BMD (C) LS-BMD (D) FA-BMD. Figure S3. Funnel plots for MR analyses of the causal effect of IBD on BMDs in replicative practice (A) TB-BMD (B) FN-BMD (C) LS-BMD (D) FA-BMD. Figure S4. Plots of “leave-one-out” analyses for MR analyses of the causal effect of IBD on BMDs in replicative practice (A) TB-BMD (B) FN-BMD (C) LS-BMD (D) FA-BMD. Figure S5. Funnel plots for MR analyses of the causal effect of UC on BMDs in initial practice (A) TB-BMD (B) FN-BMD (C) LS-BMD (D) FA-BMD. Figure S6. Plots of “leave-one-out” analyses for MR analyses of the causal effect of UC on BMDs in initial practice (A) TB-BMD (B) FN-BMD (C) LS-BMD (D) FA-BMD. Figure S7. Funnel plots for MR analyses of the causal effect of UC on BMDs in replicative practice (A) TB-BMD (B) FN-BMD (C) LS-BMD (D) FA-BMD. Figure S8. Plots of “leave-one-out” analyses for MR analyses of the causal effect of UC on BMDs in replicative practice (A) TB-BMD (B) FN-BMD (C) LS-BMD (D) FA-BMD. Figure S9. Funnel plots for MR analyses of the causal effect of CD on BMDs in initial practice (A) TB-BMD (B) FN-BMD (C) LS-BMD (D) FA-BMD. Figure S10. Plots of “leave-one-out” analyses for MR analyses of the causal effect of CD on BMDs in initial practice (A) TB-BMD (B) FN-BMD (C) LS-BMD (D) FA-BMD. Figure S11. Funnel plots for MR analyses of the causal effect of CD on BMDs in replicative practice (A) TB-BMD (B) FN-BMD (C) LS-BMD (D) FA-BMD. Figure S12. Plots of “leave-one-out” analyses for MR analyses of the causal effect of CD on BMDs in replicative practice (A) TB-BMD (B) FN-BMD (C) LS-BMD (D) FA-BMD. [file 12916_2020_1778_MOESM3_ESM.zip › Additional File3 Figure S8R2.tif]

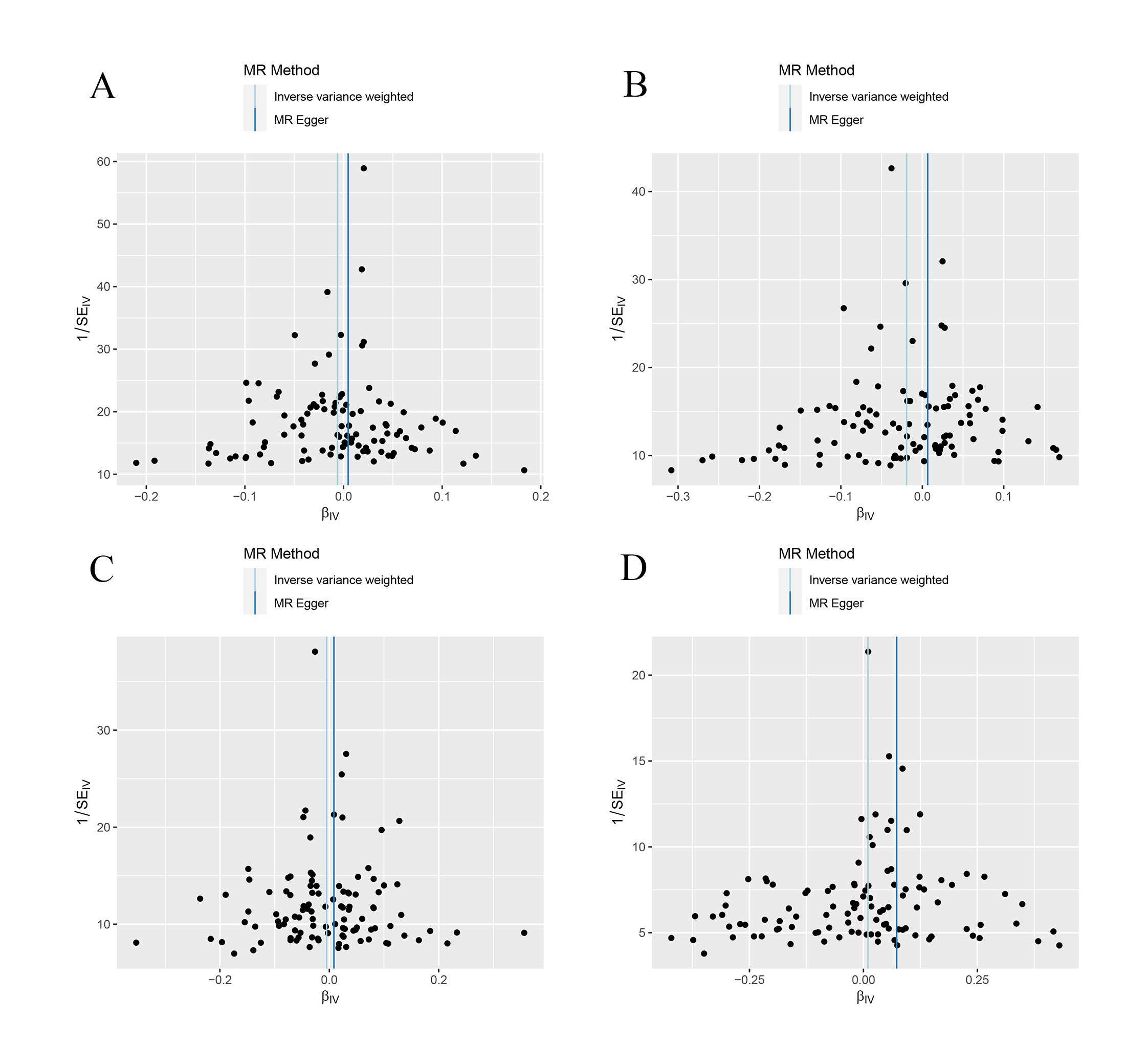

Supplement: Supplementary file 3 — Additional file 3: Figure S1. Funnel plots for MR analyses of the causal effect of IBD on BMDs in initial practice (A) TB-BMD (B) FN-BMD (C) LS-BMD (D) FA-BMD. Figure S2. Plots of “leave-one-out” analyses for MR analyses of the causal effect of IBD on BMDs in initial practice (A) TB-BMD (B) FN-BMD (C) LS-BMD (D) FA-BMD. Figure S3. Funnel plots for MR analyses of the causal effect of IBD on BMDs in replicative practice (A) TB-BMD (B) FN-BMD (C) LS-BMD (D) FA-BMD. Figure S4. Plots of “leave-one-out” analyses for MR analyses of the causal effect of IBD on BMDs in replicative practice (A) TB-BMD (B) FN-BMD (C) LS-BMD (D) FA-BMD. Figure S5. Funnel plots for MR analyses of the causal effect of UC on BMDs in initial practice (A) TB-BMD (B) FN-BMD (C) LS-BMD (D) FA-BMD. Figure S6. Plots of “leave-one-out” analyses for MR analyses of the causal effect of UC on BMDs in initial practice (A) TB-BMD (B) FN-BMD (C) LS-BMD (D) FA-BMD. Figure S7. Funnel plots for MR analyses of the causal effect of UC on BMDs in replicative practice (A) TB-BMD (B) FN-BMD (C) LS-BMD (D) FA-BMD. Figure S8. Plots of “leave-one-out” analyses for MR analyses of the causal effect of UC on BMDs in replicative practice (A) TB-BMD (B) FN-BMD (C) LS-BMD (D) FA-BMD. Figure S9. Funnel plots for MR analyses of the causal effect of CD on BMDs in initial practice (A) TB-BMD (B) FN-BMD (C) LS-BMD (D) FA-BMD. Figure S10. Plots of “leave-one-out” analyses for MR analyses of the causal effect of CD on BMDs in initial practice (A) TB-BMD (B) FN-BMD (C) LS-BMD (D) FA-BMD. Figure S11. Funnel plots for MR analyses of the causal effect of CD on BMDs in replicative practice (A) TB-BMD (B) FN-BMD (C) LS-BMD (D) FA-BMD. Figure S12. Plots of “leave-one-out” analyses for MR analyses of the causal effect of CD on BMDs in replicative practice (A) TB-BMD (B) FN-BMD (C) LS-BMD (D) FA-BMD. [file 12916_2020_1778_MOESM3_ESM.zip › Additional File3 Figure S9R2.tif]
